# Somatic ERK activation during transit amplification is essential for maintaining the synchrony of germline divisions in *Drosophila* testis

**DOI:** 10.1098/rsob.180033

**Published:** 2018-07-25

**Authors:** Samir Gupta, Bhavana Varshney, Shambhabi Chatterjee, Krishanu Ray

**Affiliations:** Department of Biological Sciences, Tata Institute of Fundamental Research, Mumbai 400005, India

**Keywords:** *Drosophila*, testis, spermatogonia, transit amplification, EGFR, ERK

## Abstract

Transit amplification (TA) of progenitor cells maintains tissue homeostasis by balancing proliferation and differentiation. In *Drosophila* testis, the germline proliferation is tightly regulated by factors present in both the germline and the neighbouring somatic cyst cells (SCCs). Although the exact mechanism is unclear, the epidermal growth factor receptor (EGFR) activation in SCCs has been reported to control spermatogonial divisions within a cyst, through downstream activations of Rac1-dependent pathways. Here, we report that somatic activation of the mitogen-activated protein kinase (Rolled/ERK) downstream of EGFR is required to synchronize the mitotic divisions and regulate the transition to meiosis. The process operates independently of the Bag-of-marble activity in the germline. Also, the integrity of the somatic cyst enclosure is inessential for this purpose. Together, these results suggest that synchronization of germ-cell divisions through somatic activation of distinct ERK-downstream targets independently regulates TA and subsequent differentiation of neighbouring germline cells.

## 1. Introduction

Appropriate generation and differentiation of the stem-cell progeny is essential for tissue regeneration and homeostasis. Often, a stem-cell progeny proliferates for a limited number of divisions before attaining terminally differentiated states [1]. These divisions, known as transit amplifications (TAs), must be regulated to maintain tissue homeostasis. Even though our understanding of stem-cell self-renewal has increased considerably [2–4], we know very little about the mechanisms regulating the TA divisions. A large part of the existing information has been obtained through experimental studies in *Drosophila* [5,6] and *Caenorhabditis elegans* [7,8]. A pioneering study in *Drosophila* testis suggested that EGFR activation in the neighbouring somatic cyst cells (SCCs), during the initial stages of spermatogonial development, helps to induce differentiation in the germline cells after four rounds of mitosis [9]. Also, elimination of the SCC lineage affected the onset of the TA in the germline stem-cell progeny [10]. This evidence highlighted the importance of cross-talk between stem-cell progeny and their neighbourhood in maintaining homeostasis.

The germline stem cells (GSCs) and the somatic cyst stem cells (CySCs) [11,12] are physically attached to a set of terminally differentiated somatic cells, called the ‘Hub’, at the apical end of *Drosophila* testis. Coordinated asymmetric divisions of a GSC and two adjoining CySCs produce a gonialblast and two SCCs, respectively. Two SCCs encapsulate a gonialblast, forming a spermatogonial cyst. A gonialblast undergoes four rounds of synchronized, TA divisions within the somatic enclosure generating a 16-cell spermatogonial cyst, which then differentiates into a 16-cell spermatocyte cyst [12]. Several cell intrinsic and extrinsic factors regulate the germline TA. The expression of germline-intrinsic factors such as Bag-of-marbles (Bam) and Benign-gonial-cell-neoplasm (Bgcn) [13–15], and signalling within the SCCs have been reported to play essential roles in regulating spermatogonial divisions and differentiation [9,16–18]. The presence of Bam in spermatogonial cells is recorded after the second mitosis (4-cell cyst), it reaches a critical threshold after the third mitosis (8-cell cyst) and disappears after the fourth mitosis (16-cell cyst) [15]. Transforming growth factor β (TGFβ) signalling regulates bam expression in early germline cells [19]. Bam is described as a necessary and sufficient factor for arresting the germline TA. Progressive accumulation up to a certain amount of Bam in the germline cells triggers TA arrest after four cycles [15,20].

Among the extrinsic factors, both EGFR [9] and TGFβ [21,22] signalling in early SCCs plays a major role in germ-cell proliferation. Germline cells secrete Spitz [23,24], an EGF-like ligand, which activates EGFR on the somatic cells [9,24]. It is conjectured that the EGFR activation progresses through Rac1 in the soma establishing proper encapsulation of the germ cells, a critical factor in the TA regulation [24]. However, the loss of somatic encapsulation during the TA stages through independent perturbations of septate junction proteins [25] did not produce excessive germline growth. Furthermore, genetic analysis has also implicated the functioning of one of the key EGFR downstream effectors, cRaf, in the soma during the TA regulation [26]. Rac/Rho and cRaf activate two separate pathways downstream to EGFR, with distinct molecular and cellular outcomes [27–30]. Therefore, it is unclear whether both cRaf-mediated downstream signalling and somatic encapsulation are involved in regulating the germline TA.

Spermatogonial nuclei have tightly packed chromatin, which is easily recognized by a relatively higher intensity of the Hoechst staining that is lowered after the transition to spermatocyte stage. Often, an empirical inspection of the population of the intense, Hoechst-stained cells at the testis apex was used to estimate the extent of germline over-proliferation in adult testis [9,24,26]. It is useful in identifying only large-scale differences. Therefore, to resolve the issues discussed above, we performed a candidate screen to identify the somatic requirements of some of the known EGFR downstream components during the germline TA using a quantitative assay [31]. We used the Gal4/UAS system to express dominant-negative (DN) and gain-of-function/constitutive-active (CA) alleles, as well as dsRNA transgenes, of EGFR downstream candidates in the SCCs during the early stage, and estimated the effects on the germline TA. We used the germ–soma ratio as an indicator of abnormal TA for the initial screen. The conclusions were further tested using appropriate secondary characterizations. The results suggest that Rolled/ERK-MAPK activation in SCCs downstream of EGFR is essential for synchronizing the germ-cell divisions within a cyst at every step during the TA. Contrary to the prevalent hypothesis, both the somatic Rac1 and integrity of cyst enclosure appeared to be inessential for the TA regulation. The somatic EGFR–ERK activity also appeared to regulate the termination of Bam expression in the germline and promote subsequent differentiation to the spermatocyte stage.

## 2. Results

### 2.1. SCC-specific functions of EGFR downstream candidates involved in regulating the germline population

**Figure 1.**
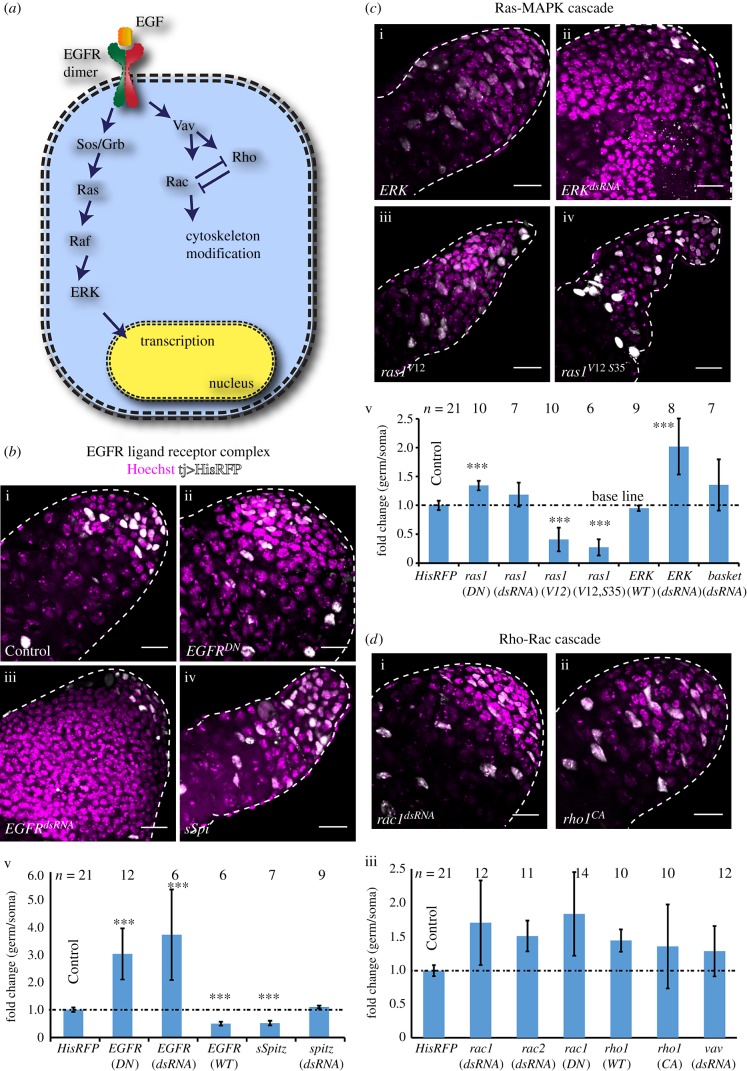
Molecular characterization of the requirement of EGFR and downstream signalling in SCCs. (*a*) Schematic illustrating the downstream components of canonical EGFR signalling. (*b*–*d*) Testes from *tjGal4/+*; *UAS-His2A-RFP/+* (Control) (*b*-i), *tjGal4/UAS-EGFR^DN^*; *UAS-His2A-RFP/UAS-EGFR^DN^* (*EGFR^DN^*) (*b*-ii), *tjGal4/+*; *UAS-His2A-RFP/UAS-EGFR^dsRNA^* (*EGFR^dsRNA^*) (*b*-iii), *tjGal4/UAS-sSpi*; *UAS-His2A-RFP/+* (*sSpi*) (*b*-iv), *tjGal4/+*; *UAS-His2A-RFP/UAS-ERK* (*ERK*) (*c*-i), *tjGal4/+*; *UAS-His2A-RFP/UAS-ERK^dsRNA^* (*ERK^dsRNA^*) (*c*-ii), *tjGal4/+*; *UAS-His2A-RFP/UAS-ras85D^V12^* (*ras1^V12^*) (*c*-iii), *tjGal4/UAS-ras85D^V12,S35^*; *UAS-His-RFP/+* (*ras1^V12 S35^*) (*c*-iv), *tjGal4/+*; *UAS-His2A-RFP/UAS-rac1^DN^* (*rac1^DN^*) (*d*-i), and *tjGal4/+*; *UAS-His2A-RFP/UAS-rho1^V14^* (*rho1^CA^*) (*d*-ii) were stained with Hoechst (magenta). His2A-RFP (white) expression marked the SCC nuclei during the TA and early spermatocyte stages. Histograms (*b*-v, *c*-v and *d*-iii) depict the relative change in the germ to somatic cell ratios in the apical part of the testes. The label below each bar indicates the transgene expressed in the SCCs by *tjGal4*. All scale bars measure 20 µm, error bars depict ±s.d., and the pairwise significance of differences (*p*-values, *<0.05, **<0.01 and ***<0.001) were estimated using one-way ANOVA^®^ and Mann–Whitney *U*-test.

We used *traffic jam-Gal4* (*tjGal4*) to express the target elements in the somatic lineage, which includes the hub, CySCs and SCCs. To assess the corresponding impact, we estimated absolute and relative changes in the germ–soma ratios (electronic supplementary material, table S1) using a quantitative assay described earlier [31]. The hub and the SCC nuclei were easily distinguished in these testes by the positions and levels of *tjGal4>His-RFP* expression (electronic supplementary material, figure S1). The expression of *EGFR^DN^* (figure 1*b*-ii) and *EGFR^dsRNA^* (figure 1*b*-iii) using the *tjGal4* driver significantly increased the germ–soma ratios (figure 1*b*-v). These testes contained highly enlarged cysts with a large number of germ cells (electronic supplementary material, table S1). Expression of the *EGFR^CA^*, wild-type EGFR and the recombinant *secretory-Spitz* (*sSpi,* figure 1*b*-iv) transgenes, respectively, reduced the germ–soma ratios significantly (figure 1*b*-v; electronic supplementary material, table S1). We also noticed significant changes in the number of germ cells and SCCs in these testes (electronic supplementary material, table S1). Previous reports indicated that the EGFR signal is not required for the CySC maintenance and division [32]. Therefore, the increase in SCCs could be attributed to the reduced proliferation of the germ cells or selective germ-cell death. *Spi* expresses in the germline cells [23]; accordingly, we found that the somatic expression of the *spi^dsRNA^* did not cause any notable alteration of the germ–soma ratio (figure 1*b*-v). Altogether, these results recapitulated the primary conclusions established by the earlier clonal analysis [9] and validated the assay for testing the roles of other candidate molecules in soma downstream of EGFR activation.

The Ras-MAPK pathway is one of the primary recipients of EGFR activation in a cell [33]. In *Drosophila*, activation of Arf-GEF [34] and Ras-cRaf-MAPK downstream of EGFR plays essential roles in various cell fate determination processes [4,35,36]. In addition, somatic loss of *cRaf* was shown to cause excessive germ-cell proliferation in *Drosophila* testis [26]. The expression of *ras85D^N17^* (*ras1^DN^*), a DN form of Ras1, increased the germ–soma ratio significantly, whereas that of *ras85D^dsRNA^* (*ras1^dsRNA^*) did not induce a significant change (figure 1*c*). A similar result was obtained with the expression of *ras64D^dsRNA^* (*ras2^dsRNA^*; electronic supplementary material, table S1, figure S2A). We reasoned that the relatively milder defects caused by the expression of *ras1^DN^* and *ras*^dsRNA^ transgenes might indicate a redundancy involving multiple Ras isoforms in these cells. This conjecture is consistent with the observation that somatic expression of *ras85D^V12^* and *ras64D^V12^* produced similar phenotypes (electronic supplementary material, table S1). We found that the expression of *rolled/ERK^dsRNA^* (*ERK^dsRNA^*, figure 1*c*-ii) increased the germ-cell population significantly (figure 1*c*-v), whereas that of wild-type *ERK* (figure 1*c*-i) did not alter the germ–soma ratio. Together, these results suggested that the ERK activation is tightly regulated. Also, expressions of the constitutively active *ras85D^V12^* (*ras1^CA^*, figure 1*c*-iii) and *ras85D^V12, S35^* (*ras1^V12 S35^* figure 1*c*-iv), which selectively activate the ERK-MAPK [37,38], as well as *ras64D^V12^* (*ras2^V12^*; electronic supplementary material, table S1, figure S2A), reduced the germ–soma ratios (figure 1*c*-v). The *ras1^V12, S35^* expression induced relatively more significant reduction of the germline cells than that of the *EGFR* and *sSpi* (electronic supplementary material, table S1). Thus, Ras-MAPK appeared to play a significant role in regulating the germline proliferation.

A previous study reported that somatic expression of the *rac1^DN^* and *vav^dsRNA^* transgenes induce strong germline over-proliferation along with disruption of the somatic enclosure, and that of *rho1^DN^* suppressed the phenotype, in the conditional *spi* mutant (*spi^77–20^*) background [24]. The *tjGal4*-mediated somatic expression of *rac1^dsRNA^* (figure 1*d*-i) resulted in a marginal increase in the germ–soma ratios (figure 1*d*-iii). A similar result was obtained due to the coexpression of both *rac1^dsRNA^* and *rac2^dsRNA^* (electronic supplementary material, table S1). Expression of wild-type *rho1*, *rho1^CA^* and *vav^dsRNA^*, which are expected to downregulate Rac activity in these cells, did not alter the germ–soma ratios (figure 1*d*-iii), whereas that of *rac1^CA^* or *rho1^dsRNA^* eliminated the testis. We confirmed that somatic expression of the *rac1^dsRNA^* could effectively eliminate GFP-Rac1, expressed through an endogenous promoter, from the SCCs (electronic supplementary material, figure S2B). However, it did not induce abnormal cyst accumulation at the testis apex. Also, we noted that the somatic expression of *EGFR^dsRNA^* did not disrupt GFP-Rac1 localizations around the boundary of over-proliferated cysts (electronic supplementary material, figure S2B). Furthermore, coexpression of both the *rac1^dsRNA^* and *rac2^dsRNA^* in the SCCs did not alter the cyst distribution profile (electronic supplementary material, figure S2C). Altogether, these selective perturbations of known EGFR downstream molecules in SCCs highlighted a specific role of Rolled/ERK-MAPK-dependent processes in regulating the germline TA. Although it appeared to have a requirement in testis development or maintenance, the manipulations of somatic Rac activity during the early stages did not alter the germline population significantly.

### 2.2. EGFR activates Rolled/ERK in the SCCs

**Figure 2.**
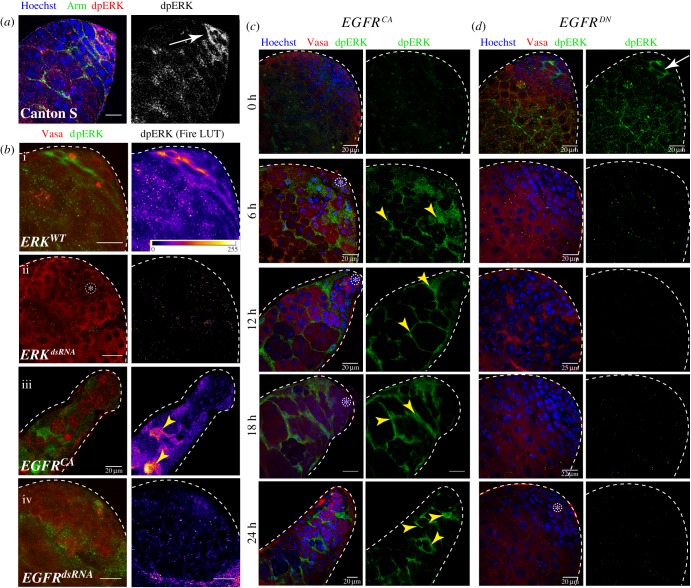
Correlation of somatic ERK phosphorylation with EGFR activation and downregulation. Adult testes from different genotypic backgrounds as indicated in the panels were stained with anti-Arm (green), anti-dpERK (red) and the Hoechst (blue) dye (*a*), or, anti-dpERK (green), anti-Vasa (red) and Hoechst (magenta) dye (*b–d*). The UAS-transgenes indicated on the panels were expressed in the SCCs either by using *tjGal4* (*b*), or *tjGal4* along with *tub-Gal80^ts^* (*c*,*d*). In the latter case, the expression level was progressively elevated by growing the stocks at 29°C for different extents as indicated on the left margin of panel (*c*). The images depicting anti-dpERK staining in panels (*b*) and (*c*) were thresholded to the 95% intensity values to highlight the peak staining for an effective visual comparison. Arrowheads indicate intense cytoplasmic anti-dpERK staining in the SCCs. Scale bars measure 20 µm.

EGFR activation phosphorylates two tyrosine residues on ERK through the Ras-MAPKinase cascade [39], which is estimated by immunostaining with the dpERK-specific monoclonal antibody mAb4370 [40]. Previous studies reported widespread dpERK staining in the SCCs during the TA stages [41]. We found a somewhat patchy dpERK staining pattern using mAb4370 (figure 2*a*). *ERK^dsRNA^* expression in the SCCs eliminated the staining confirming its specificity (figure 2*b*-ii). The *EGFR^CA^* expression, on the other hand, enhanced the dpERK staining (figure 2*b*-iii), and the *EGFR^dsRNA^* expression eliminated the staining (figure 2*b*-iv). These results indicated that the EGFR signalling in the SCCs activates ERK, which is also the dominant pathway regulating the process. To further determine a causal relationship between the ERK phosphorylation and the dose of EGFR signalling, we calibrated the levels of *EGFR^CA^* expression in the SCCs using the *Gal4/Gal80^ts^* system (figure 2*c*). Increasing duration of growth at 29°C progressively increased the dpERK staining in the SCCs in *tjGal4/UAS-EGFR^CA^*; *tub-Gal80^ts^/+* testes (figure 2*c*), suggesting that progressive increase in the somatic activation of EGFR proportionately enhances the levels of ERK phosphorylation. A converse experiment, performed using the *tjGal4/UAS-EGFR^DN^*; *tub-Gal80^ts^/+* stock further showed that the loss of somatic EGFR activity reduced the ERK phosphorylation below detection levels from 6 h onwards (figure 2*d*). Together, these results suggested that EGFR signalling in SCCs activates the ERK-MAPK downstream, which is also consistent with earlier reports [41].

### 2.3. EGFR and ERK inactivation in SCCs deregulates spermatogonial TA independent of bam expression

**Figure 3.**
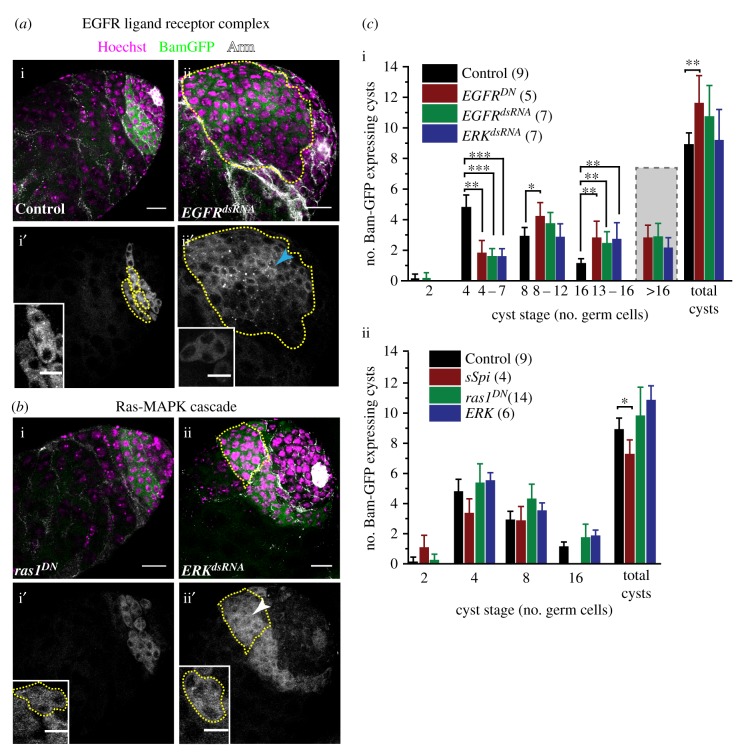
Loss of EGFR signalling in SCCs deregulates the termination of *bam* expression in the germline cells. (*a*,*b*) Testes from *tjGal4 bamp-BamGFP/+*; *UAS-His2A-RFP/+*(Control) (*a*-i), *tjGal4 bamp-BamGFP/+*; *UAS-His2A-RFP/UAS-EGFR^dsRNA^* (*EGFR^dsRNA^*) (*a*-ii), *UAS-ras85D^DN^/Y*; *tjGal4 bamp-BamGFP/+*; *UAS-His2A-RFP/+* (*ras1^DN^*) (*b*-i), and *tjGal4 bamp-BamGFP/+*; *UAS-His2A-RFP/UAS-ERK^dsRNA^* (*ERK^dsRNA^*) (*b*-ii) were stained with anti-Arm (white) marking the cyst boundary and Hoechst 33342 (magenta). BamGFP (green) fluorescence marked the bam-expressing stages. (*a*-i′,ii′, *b*-i′,ii′) Grey LUT of BamGFP fluorescence in the testes and magnified views of 4-cell cysts (insets) highlight the Bam levels in individual germ cells within cysts in different genetic backgrounds. (*c*) Histograms depict mean (±s.d.) *bam-*expressing cysts in different genotypic backgrounds. In the *EGFR* and *ERK* backgrounds, often the cyst boundaries were not clear. Therefore, they are enumerated in a range bound manner. All scale bars, error bars and statistical significance values are applied according to the description in figure 1. Inset scale bars measure 10 µm.

The level of Bam expression in germline cells is reported to play a critical role in arresting the spermatogonial TA [42]. Loss of Dynein and Myosin V in the SCCs, as well as TGFβ signalling, downregulated Bam expression and induced germline over-proliferation [18,43]. To understand whether the loss of ERK activity could delay the onset of Bam expression during the 1–16 cell stages, we studied the *bamp-BamGFP* expression pattern, which reports the endogenous *bam* expression levels [42], in the *EGFR^DN^ EGFR^dsRNA^* and *ERK^dsRNA^* backgrounds (figure 3*a*,*b*). Although BamGFP marked several cysts in these testes, the distribution of GFP fluorescence in enlarged cysts was visibly inhomogeneous (figure 3*a*-ii′, *b*-ii′). Also, occasional cysts in the mutant backgrounds appeared to contain unusual (other than 4, 8 or 16) numbers of Bam-positive cells. An irregular Armadillo/β-catenin (Arm) staining (discussed in detail later) further confounded the cyst categorization. The ambiguity levels were higher in the 8–16 cell categories. Therefore, we classified BamGFP cysts in the mutant backgrounds under four broad classes: 4–7, 8–12, 13–16 and greater than 16 cells, respectively. There was a significant reduction in the number of 4–7 cells, and an increase in the number of 8–12, 13–16 and greater than 16-cell cysts in these testes (figure 3*c*-i). In comparison, the ectopic activation of EGFR through somatic *sSpi* overexpression marginally reduced the total number of BamGFP-expressing cysts and had no 16-cell cysts (figure 3*c*-ii). Somatic expression of *ras1^DN^* and wild-type *ERK* transgene (figure 3*b*-i′, *c*), as well as *rac1^dsRNA^* and *rho^CA^* (electronic supplementary material, figure S3A), did not alter the BamGFP pattern and the cyst sizes were the same as the wild-type.

Loss of BamGFP-positive cysts in the 4–7 cell category and a corresponding increase of the 8–16 and greater than 16-cell populations in the EGFR and ERK loss-of-function backgrounds were intriguing. It could suggest that the loss of EGFR activation could delay the onset of Bam expression, which usually commences after the second mitosis [15]. Consistent with this hypothesis, several proliferative germline cells were found in the apical region of testes in the mutant backgrounds (electronic supplementary material, figure S3B), which is usually occupied by the 4-cell, Bam-positive cysts in the wild-type testis. Owing to irregular Arm staining, it was difficult to ascertain whether BamGFP marked all the spermatogonial cysts in these mutant testes. By contrast, the gain of somatic EGFR activity in the *sSpi* overexpression background did not increase the pool of 2-cell cysts marked by BamGFP significantly. Together, these results indicated that EGFR/ERK activation in the soma is not required to induce Bam expression. However, it may play a role in attenuating Bam level after the fourth mitotic cycle as indicated by the absence of 16-cell cysts marked by BamGFP in the *sSpi* background.

### 2.4. Somatic downregulation of EGFR disrupts synchronized spermatogonial divisions

**Figure 4.**
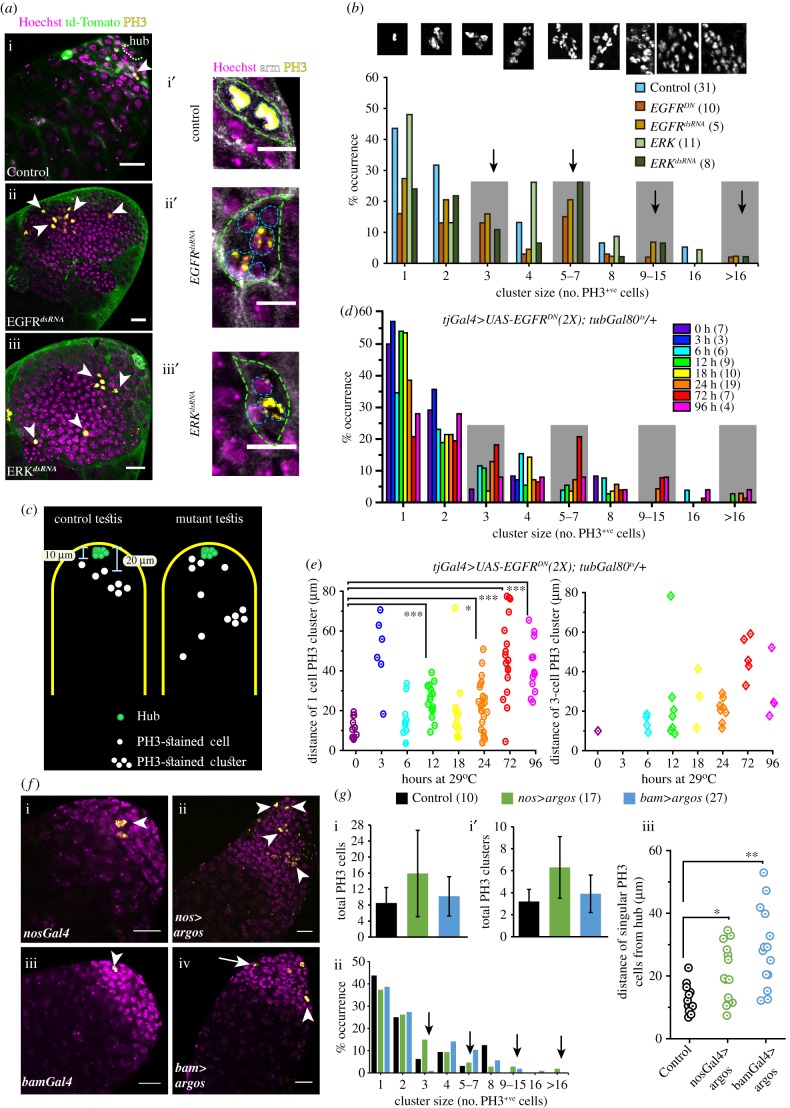
Disruption of EGFR signalling in SCCs affects the synchrony of germline divisions during TA. (*a*) Testes from *tjGal4 UAS-tdTomato/+* (Control) (a), *tjGal4 UAS-tdTomato/+*; *UAS-EGFR^dsRNA^/+*(*EGFR^dsRNA^*) (b) and *tjGal4 UAS-tdTomato/+*; *UAS-ERK^dsRNA^+*(*ERK^dsRNA^*) (c) were stained with anti-PH3 (yellow) and the Hoechst dye (magenta). Arrowheads point to M-phase nuclei. (i′–iii′) Enlarged views of 2–4-cell cysts from the wild-type control (i′) and mutant (ii′,iii′) testes that were stained with the Hoechst dye (magenta), anti-Arm (white) and anti-PH3 (yellow). Cyst boundaries (dashed green) and germ cells (dashed cyan) are marked. (*b*) Histograms depict relative, size-wise distribution of PH3-stained clusters in different genetic backgrounds. Shaded boxes and arrows highlight odd-sized clusters. (*c*) A schematic depicts the typical distribution of PH3-stained cells and clusters along the length of the testis. The control represents a summary of observation from *tjGal4/UAS-His-RFP,* and the mutant summarizes the observations made in the *EGFR^DN^* backgrounds as shown in panel (*e*). (*d*) Histograms depict relative distributions of odd and even-sized PH3-stained clusters with decreasing dose of EGFR activity in SCC. (*e*) Cluster plots depict distance of 1- and 3-cell PH3-stained clusters from the apical tips of testes. (*f*) Testes from *nosGal4vp16* (i), *UAS-argos/+*; *nosGal4vp16/+*(*nos>argos*) (ii), *bamGal4vp16* (iii), and *bamGal4vp16/UAS-argos* (*bam>argos*) (iv), were stained for PH3 (yellow) and with the Hoechst dye (magenta). (*g*) Histograms depict mean (±s.d.) (i,i′) and relative, size-wise, distributions (ii) of PH3-stained clusters obtained from the control (*nosGal4vp16*) and two different mutant backgrounds. (iii) Scatter plots depict distances of singular PH3-stained cells from the testis apex in different backgrounds. All scale bars, error bars and statistical significance values are applied according to the description in figure 1. Scale bars for *a*-i′, *a*-ii′ and *a*-iii′ measure 10 µm.

The germline cells within a cyst divide in synchrony producing 2-, 4-, 8- and 16-cell cohorts. Therefore, the occurrence of irregular-size cysts observed in the EGFR and ERK loss-of-function backgrounds could arise due to the asynchronous division of germline cells within a cyst. To test the effects of EGFR downregulation on germline mitosis, we stained the testes with phosphohistone-3 (PH3) antibody which marks the M-phase nuclei. As shown before [41], PH3-stained cells always appeared in clusters of 2, 4, 8 and 16, in wild-type testes, indicating that the germline cells of a cyst always divide together (figure 4*a*,*b*). The staining always marked all the cells within a cyst (figure 4*a*-i′). Somatic overexpression of *EGFR^DN^*, *EGFR^dsRNA^* or *ERK^dsRNA^* increased the number of PH3-stained germ cells significantly, whereas overexpression of *rac1^dsRNA^* or *rho1^CA^* caused no significant change (electronic supplementary material, figure S4A–F and G–G′). The PH3 staining in both the small (figure 4*a*-ii′ and iii′) as well as the unusually large cysts (arrowheads, figure 4*a*-ii and iii), was sporadic in the *EGFR^DN^*, *EGFR^dsRNA^* and *ERK^dsRNA^* backgrounds. We also noted isolated PH3-stained cells further away from the hub than usual (figure 4*c*), and incomplete PH3 staining within some of the small-size (4–8 cell) cysts (electronic supplementary material, figure S4I) in these backgrounds. An estimation of PH3-stained clusters in these testes suggested that the somatic loss of EGFR and ERK activity could substantially increase the proportions of odd (3, 5–7, 9–15 mitotic cells) clusters, implying that the somatic EGFR activity is needed to synchronize the germline mitoses within a cyst. A majority of these odd clusters were located within the abnormally large (greater than 16 cell) cysts. Once again, due to irregular Arm staining in the mutant testes, often it was difficult to assess the class of cysts carrying an irregular number of PH3-stained cells and whether disjointed PH3-stained cells/clusters were part of a single cyst.

Therefore, we devised an unbiased assay. We noted that the GSCs and gonialblasts appeared close to the hub in both wild-type (8.2 ± 1.8 µm and 15.9 ± 2.4 µm, *N* = 7, *n* = 14) and *EGFR^DN^* (9.6 ± 1.2 µm and 15.6 ± 2.3 µm, *N* = 7, *n* = 14) backgrounds after 4 days at 29°C. The cysts carrying 2 cells or more appeared further away. Thus, single PH3-positive cells, indicative of GSC or gonialblast divisions, are expected to appear within 18–19 µm from the hub, and greater than 2-cell clusters would be further away. To ascertain whether somatic loss of EGFR–ERK activity could desynchronize mitoses in 2–16 cell stages, we estimated the distances of the single cell and 3 cell, PH3-stained clusters from the hub. The EGFR activity was progressively reduced by growing *tjGal4>EGFR^DN^ (2x)*; *tub-Gal80^ts^* flies at 29°C for extended durations. The number of odd-sized clusters progressively increased with increased growth durations at 29°C (electronic supplementary material, figure S5A-B; figure 4*d*), indicating a direct correlation between the loss of EGFR activity and asynchrony. The analysis further suggested that greater than 16-cell PH3-stained clusters, a definitive indicator of post-TA deregulation, appeared from 12 h onwards (figure 4*d*), and Arm staining was recognizable until 18 h of growth at the non-permissive temperature (electronic supplementary material, figure S5A). Together, these observations helped to clarify that the unusually large cysts were formed after 12 h. Interestingly, we found several isolated PH3-stained cells further away than usual, from 3 h onwards, in the *tjGal4>EGFR^DN^ (2x)*; *tub-Gal80^ts^* testes (figure 4*e*). Increasing expression of EGFR^DN^ in the somatic cells also generated several atypical 3-cell clusters within the 15–20 µm range from 6 h onwards (figure 4*e*). The testes did not contain any unusually large (greater than 16 cell) cysts at these stages. Together, these analyses suggested that the loss of EGFR activity disrupts the synchrony of germline divisions within a cyst during TA (4–16 cell stages).

The expression of the EGF antagonist Argos (*aos*), using either *nosGal4* (figure 4*f*-ii) and *bamGal4* (figure 4*f*-iv), is expected to reduce EGFR activations locally during 1–2 cell and 4–16 cell stages, respectively. Therefore, we used it as an independent means to ascertain the local role of the somatic EGFR activation on the TA synchronization. Expression of the *nosGal4>aos* and *bamGal4>aos* produced several isolated PH3-stained cells (figure 4*g*-i,ii) at positions beyond the expected range (figure 4*g*-iii). Most importantly, the expression of *bamGal4>aos* produced a large number of scattered PH3-stained cells farther away from the hub than the wild-type control. Altogether, these results indicated that EGFR–ERK activation in SCCs is required at every TA-division cycle to synchronize the germ-cell divisions within a cyst. Out of the 101 PH3-stained clusters observed in various control testes, we found only one 3-cell and one 5-cell cluster. These occurrences are considered to be caused by incomplete immunostaining or subjective bias. For some inexplicable reason, we did find a few 3-cell (approx. 5%, 2/42) and 5–7-cell (approx. 2.5%, 1/42) clusters in the *bamGal4* background (figure 4*g*-ii), which is perhaps influenced by the genetic background and small size of the cohort.

### 2.5. Somatic loss of EGFR activity affects Armadillo localization on the cyst membrane

**Figure 5.**
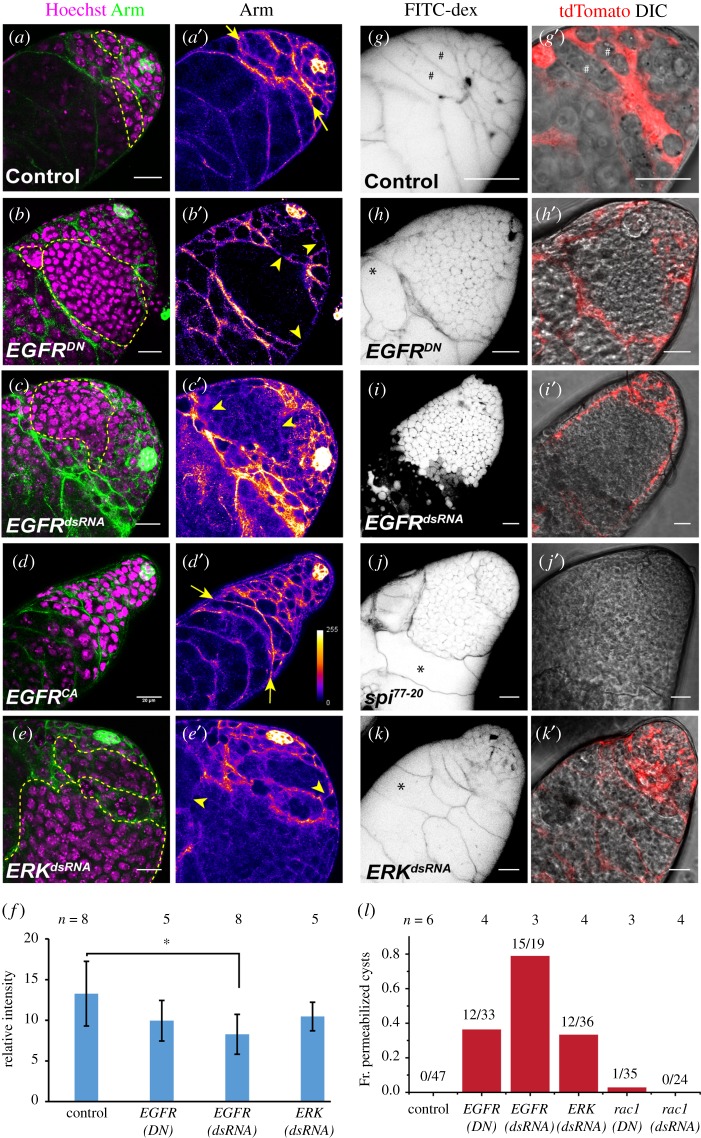
Loss of EGFR activity in the SCCs reduces Armadillo localization to the cyst membrane. (*a*–*e*) Testes from *tjGal4/+*; *UAS-His2A-RFP/+* (Control) (*a*), *tjGal4/UAS-EGFR^DN^*; *UAS-His2A-RFP/UAS-EGFR^DN^* (*EGFR^DN^*) (*b*), *tjGal4/+*; *UAS-His2A-RFP/UAS-EGFR^dsRNA^* (*EGFR^dsRNA^*) (*c*), *tjGal4 UAS-His2A-RFP/+*; *UAS-EGFR^CA^/+* (*EGFR^CA^*) (*d*) and *tjGal4 UAS-His2A-RFP/+*; *UAS-ERK^dsRNA^/+* (*ERK^dsRNA^*) (*e*), were stained with anti-Arm (green) and the Hoechst dye (magenta). (*a*′–*e*′) anti-Arm staining is illustrated in pseudo-colour LUT. (*f*) Histograms depict average Arm intensity (relative) in different genetic backgrounds. (*g*–*k*) Testes from *tjGal4/+*; *UAS-tdTomato/+* (Control) (*g*), *tjGal4/UAS-EGFR^DN^*; *UAS-tdTomato/UAS-EGFR^DN^* (*EGFR^DN^*) (*h*), *tjGal4/+*; *UAS-tdTomato/UAS-EGFR^dsRNA^* (*EGFR^dsRNA^*) (*i*), *spi^77–20^* (*j*) and *tjGal4/+*; *UAS-tdTomato/UAS-ERK^dsRNA^* (*ERK^dsRNA^*) (*k*) were incubated in FITC-dextran (inverted LUT). (*g*′–*k*′) tdTomato expression (red) in SCC and DIC overlay. (*l*) Histograms depict the distribution of cysts penetrated by FITC-dextran in different genetic backgrounds. All scale bars, error bars and statistical significance values are applied according to the description in figure 1.

Several reports have highlighted the importance of the quality of somatic encapsulation around transit amplifying spermatogonia. For example, elimination of CySCs resulted in over-proliferation of early germline cells and spermatogonia [10]. Also, Rac1 activation downstream of EGFR has been implicated in the maintenance of somatic encapsulation and the TA regulation [23,24]. Arm localizes along the membrane of both the SCCs and the hub cells (figure 5*a*,*a*′). Somatic downregulation of EGFR (figure 5*b*,*c*) and ERK (figure 5*e*) activities disrupted the Arm staining. It appeared discontinuous around both the regular sized and overpopulated cysts (yellow arrowheads, figure 5). Estimation of the total Arm staining in the TA region, normalized by the staining intensity in the hub, also revealed small but significant reduction due to EGFR knockdown (figure 5*f*). These results suggested that though the cyst encapsulation is occasionally disrupted due to the somatic loss of EGFR activity, Rac1 is not involved in the process (electronic supplementary material, figure S6).

FITC-dextran dye permeation assay was used to test the integrity of SCC encapsulation [25]. In wild-type testes, the dye permeates early stage cysts (GSCs, 1- and 2-cell stages), but failed to access the later stages (#-marked cysts, figure 5*g*,*g*′). The larger cysts, produced due to the somatic downregulation or loss of EGFR, were permeant to FITC-dextran (figure 5*h*,*i*), even though tdTomato expression in SCC indicated an intact encapsulation (figure 5*h*′,*i*′). Loss of EGF in the testis (form *spi^77–20^* adults grown at 29°C for 4 days) also produced similar dye penetration defect (figure 5*j*,*j*′), suggesting that the somatic EGFR activity is required for either formation or maintenance of such a permeability barrier around spermatogonia. Interestingly, we noticed that a fraction of the abnormally large (greater than 16 cells) cysts was not permeant to the dye in the *EGFR^DN^* and homozygous *spi^77–20^* backgrounds (*-marked cysts, figure 5*h* and *j*). Also, somatic knockdown of ERK did not result in a definite breach of the somatic cyst encapsulation even in the enlarged cysts (*-marked cysts, figure 5*k*). These observations indicated that the integrity of the cyst encapsulation might not be essential for regulating the germline TA. Also, somatic overexpression of the *Rac1^dsRNA^* and *Rho1^CA^* did not induce aberrant dye permeation (electronic supplementary material, figure S6). Thus, we concluded that somatic Rac/Rho pathway would be redundant for maintaining the somatic cyst enclosure.

### 2.6. The integrity of somatic encapsulation is redundant for the TA regulation

**Figure 6.**
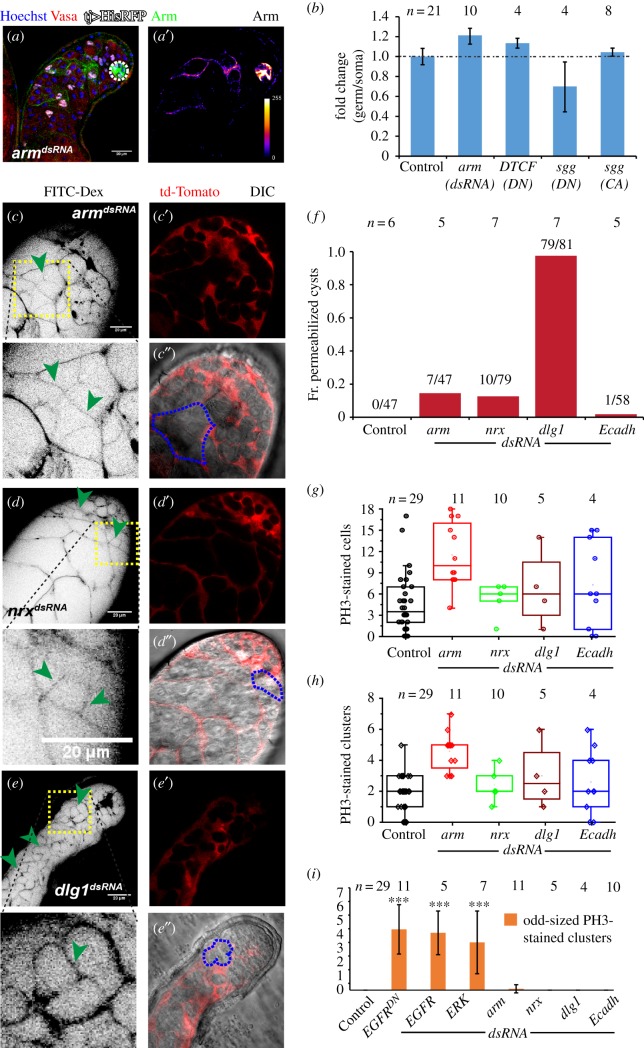
Role of somatic encapsulation in maintaining the germline TA. (*a*) Testes from *tjGal4/+*; *UAS-arm^dsRNA^/UAS-His2A-RFP* (*arm^dsRNA^*) males were stained with anti-Arm (green) and the Hoechst dye (magenta). His2A-RFP (white) expression marked the SCC nuclei during the TA. (*a*′) anti-Arm staining is depicted in pseudo-colour LUT. (*b*) Histograms depict fold change in the germ–soma ratios in His-RFP (Control), *tjGal4/+*; *UAS-arm^dsRNA^/UAS-His2A-RFP* (*arm^dsRNA^*) and *tjGal4/+*; *UAS-DTCF^DN^/UAS-His2A-RFP* (*DTCF^DN^*) backgrounds. (*c*–*e*) Testes from *tjGal4/+*; *UAS-tdTomato/UAS-arm^dsRNA^* (*arm^dsRNA^*) (*c*), *tjGal4/+*; *UAS-tdTomato/UAS-nrx^dsRNA^* (*nrx^dsRNA^*) (*d*) and *tjGal4/+*; *UAS-tdTomato/UAS-dlg1^dsRNA^* (*dlg1^dsRNA^*) (*e*) were dissected in Schneider's medium and incubated in FITC-dextran (inverted LUT). Green arrowheads point to the dye-permeated cyst. (*c*′,*c*″, *d*′,*d*″ and *e*′,*e*″) tdTomato expression (red) in SCC and DIC overlay, magnified cysts are outlined by dashed blue lines. Scale bars measure 20 µm. (*f*) Histograms depict the distribution of cysts penetrated by FITC-dextran in different genetic backgrounds. (*g*,*h*) Dot plots depict PH3-stained cells (M-phase index) (*g*) and PH3-stained clusters (*h*) in different genetic background adult testes. (*i*) Histograms depict the average odd-sized (desynchronized) PH3-stained clusters across various genetic backgrounds. All error bars depict ±s.d., and the pairwise significance of differences (*p*-values, *<0.05, **<0.01 and ***<0.001) were estimated using one-way ANOVA^®^ and Mann–Whitney *U*-test.

Armadillo/β-catenin is a component of the canonical Wnt-signalling cascade [44]. It has been implicated in the formation of adherence junctions [45]. The presence of Arm along the cyst contour suggested that adherens junctions could participate in the formation and maintenance of the somatic encapsulation. Wnt signalling and downstream components have been shown to regulate GSCs in *Drosophila* ovary [46,47] and testis [48]. It has been reported that Wnt signalling is extrinsically required for the differentiation of GSC progeny in *Drosophila* ovaries [49]. Therefore, we tested the requirement of Arm in SCC encapsulation and that of Wnt signalling downstream in the SCCs. Expression of *arm^dsRNA^* in the soma resulted in total loss of Arm staining in almost 50% of cases (figure 6*a,a*′), and these testes had relatively fewer elongated cysts. However, the somatic overexpression of *arm^dsRNA,^ sgg^CA^, sgg^DN^* and *DTCF^DN^*, which is expected to perturb the Wnt-signalling cascade, did not affect the germ–soma ratio at the apical tip of the testes (figure 6*b*; electronic supplementary material, table S2). Also, the somatic overexpression of Arm^S10^, a relatively stable form of Arm [50], did not alter the germ–soma ratio, and the total number of spermatogonial cysts were not significantly changed (electronic supplementary material, figure S7A), indicating that the canonical Wnt cascade is redundant in regulating the germline TA.

Interestingly, loss of Arm/β-catenin disrupted the somatic permeability barrier around the germline cysts as revealed by the FITC-dextran permeability assay (figure 6*c*,*c*′). These testes had a visibly lower number of elongated and mature spermatids (electronic supplementary material, figure S7C), suggesting a block in further differentiation beyond the spermatocyte stage. To test the role of EGFR activity on Arm functions in the SCCs, we overexpressed Arm^S10^ in SCCs in the *EGFR^DN^* (2×) and *ERK^dsRNA^* background (electronic supplementary material, figure S7D,E). It did not rescue the asynchronously dividing overgrown germline phenotype, further indicating that the germline TA defect is not linked to the loss of Armadillo at the SCC membrane. Various septate junction components, such as Neurexin IV (NrxIV) [51] and Discs-large-1 (Dlg1), localize on early SCC membrane [25]. The SCC-specific knockdown of NrxIV and Dlg1 disrupted the somatic permeability barrier (figure 6*d*–*f*). The loss of Dlg1 severely affected the spermatid differentiation. However, the mitotic indices were unaltered (figure 6*g*,*h*), and there were no odd-sized PH3-stained clusters in these testes (figure 6*i*). These results further corroborated that the integrity of the cyst encapsulation is not essential for progression and termination of germline TA.

## 3. Discussion

### 3.1. The molecular mechanism of EGFR downstream activation in SCCs

**Figure 7.**
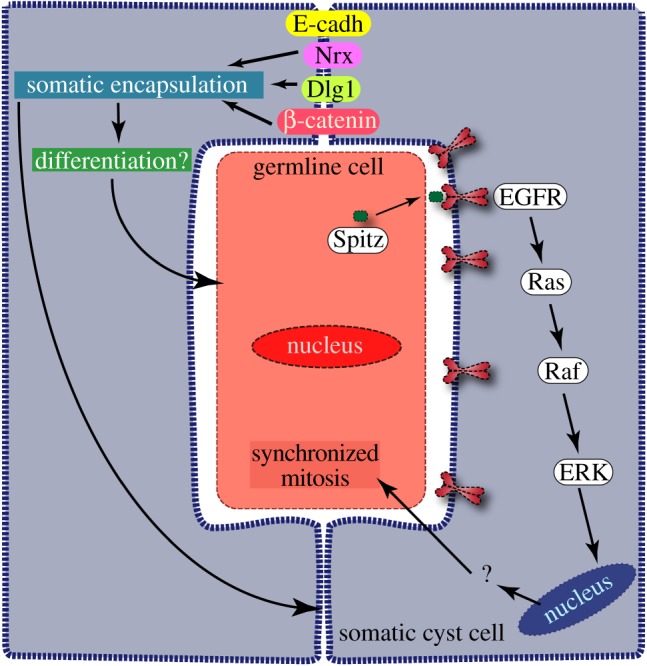
Schematic summary of the results and conclusions. Secretion of Spitz, an EGFR ligand, form the germline cells [23] activates EGFR on the somatic cyst cell membrane [9]. It induces the phosphorylation of the MAPkinase, ERK, which in turn helps to synchronize the germline divisions within a cyst and facilitates the transition to the spermatocyte stage. Cell adhesion proteins β-catenin, Discs-large-1 (Dlg1) and Neurexin IV (Nrx) help to form a tight somatic enclosure around the germline cyst that is essential for differentiation after the spermatocyte stage.

The results described above provide experimental verifications of two critical conjectures regarding the regulation of germline TA: the molecular signalling downstream of the somatic EGFR involved in the germline TA regulation and the role of somatic encapsulation. Estimation of germline and somatic cell ratios allowed us to identify the role of EGFR downstream components in regulating the germline proliferation (figure 7). The estimation also revealed subtle quantitative differences in the involvement of the candidate genes/gene products in this process. For example, we found that loss of EGFR in SCCs produced a more robust phenotype as compared to that of ERK. Thus, it indicated that additional EGFR downstream might also be involved in the TA regulation other than the ERK-dependent processes. Perturbing two of the known canonical EGFR downstream components, the Ras GTPases and ERK-MAPK, produces different effects on the germline growth and differentiation. It appeared that Ras1 and Ras2 redundantly act in SCCs for regulating the germline divisions, whereas the ERK loss generated a severe over-proliferation defect similar to that of the EGFR. Previous studies indicated cRaf activity in SCCs is also essential in regulating the germline proliferation [26]. Therefore, we concluded that the ERK activation downstream of EGFR could occur through a rather direct process. Alternatively, the Ras knockdown in SCCs was inadequate for attenuating the ERK activation below the required levels. These possibilities are needed to be tested by further direct experimental analysis in future.

### 3.2. The ERK-MAPK is the primary target of EGFR activation in SCCs which synchronizes the germline divisions through a novel mechanism

The somatic activation of the novel EGFR-ERK pathway synchronizes germline mitoses within a cyst. Although it was noted earlier in the overgrown cysts [41], the results described in this manuscript established a tight correlation between the EGFR-ERK activation in SCCs and synchronization of germline divisions in the cyst. Loss of this synchrony due to inadequate ERK activation in SCCs led to abnormal cellular growth within a cyst and blocked further differentiation of the germline cells. These observations suggested that the EGFR/ERK activity in SCCs provides a signal to the encapsulated germline for either triggering the mitoses or managing the checkpoint control systems. The canonical Ras-ERK activation is known to propagate through the ETS-domain containing transcriptional activators. In *Drosophila*, the ETS-family member is coded by *pointed* (*ptd*). A preliminary study suggested that both *ptd-lacZ* and *ptdGal4>eGFP* do not express in the adult testis, and expression of *UAS-ptd^dsRNA^* by *tjGal4* did not induce the germline proliferation defect (S. Gupta, B. Varshney and K. Ray 2014, unpublished data). Both the phospho-inositol-3-kinase (PI3K)-dependent processes [52,53] and glycogen-synthase-kinase-3β (GSK3β) activity [54] were implicated in the ERK activation and cell proliferation in different cell types. In a previous report, we showed that the somatic PI3K activity is inessential for regulating the TA divisions [31]. Here, we have further shown that somatic perturbations of Sgg/GSK3β did not alter the germline mitosis. Therefore, studies in future need to be focused on determining the somatic requirements of other ERK targets for their role in the TA regulation process. The current study helped to highlight this unique feature of somatic EGFR function employed during the germline TA.

The level of Bam in germline cells, which appears at the 4-cell stage and peaks at the 8-cell stage, limits the mitotic divisions to four rounds under wild-type conditions [15]. However, its role in synchronizing the germline divisions was not tested. We ruled out the possibility that loss of the synchrony could be resulted due to non-uniform levels of Bam expression in the germline in EGFR-ERK loss-of-function backgrounds. The BamGFP levels were nearly uniform in the 4–16-cell cysts. We only observed the non-uniform distribution of BamGFP in greater than 16-cell cysts. A previous study inferred that the *bam* expression could be delayed in the germline cells in the EGFR mutant background [9]. Although the possibility could not be ruled out, we did not find significant evidence to support the argument. Instead, the results indicated that the somatic activity of EGFR-ERK would be required to terminate the Bam expression at an appropriate stage. The loss of somatic cyst stem cells and the integrity of the cyst enclosure was suggested to desynchronize the germline divisions [10,24]. We found that somatic loss of Arm/β-catenin, and that of the septate junction proteins NrxIV and Dlg1, could independently disrupt the integrity of somatic cyst enclosure. However, it did not induce asynchronous and excessive germ-cell proliferation. These observations are consistent with a previous report which indicated that the loss of NrxIV in the SCCs only affects the differentiation after the spermatocyte stage [25].

### 3.3. Rho/Rac signalling in SCCs and quality of cyst encapsulation do not regulate the TA

Various small GTPases have been implicated in tumour progression as they modify the cytoskeleton inducing cell-shape changes and cell migration [28,55–57]. Rho GTPases act downstream to many different growth factor-mediated signalling cascades [58]. Also, they regulate the focal adhesion kinases [28] influencing actin and microtubule modification. Among various other small GTPases, the role of Rac and Rho GTPases has been previously reported to be antagonistic to each other [24,28]. EGFR-dependent Rac1 activation in SCCs is suggested to play an essential role in controlling the germline proliferation and differentiation within a cyst [24]. We found that perturbation of Rac1 and 2, as well as Rho1, functions in SCCs does not affect the somatic encapsulation and the TA divisions. Independent disruption of the integrity of cyst encapsulation by loss-of-function backgrounds of various junction molecules did not enhance or desynchronize the germline divisions. Further, we found that the cyst enclosure was intact in several over-proliferated cysts in the EGFR loss-of-function backgrounds. Finally, the perturbation of Rac GTPases in SCCs did not disrupt the cyst encapsulation as conjectured before. These results helped to discount a role of cyst encapsulation in regulating the germline TA.

Altogether, the experimental data described here eliminated two critical conjectures regarding the mechanism of TA regulation and helped to focus the attention back to the downstream targets of ERK in this process. To further understand the mechanism, we would now have to look for both the known and novel targets of ERK in the SCC for their role in germline proliferation. The EGFR-ERK pathway is known to induce expression of several miRNAs [59] and TGFβ ligands [60,61]. Previous studies have indicated that one of the TGFβ ligands, *glass-bottom-boat* (*gbb*), is involved in regulating the germline proliferation [22,43]. One or more of these factors could be involved in providing the feedback to the germline. Alternatively, a multi-factorial process influencing the physical environment within a cyst, miRNAs and other signalling factors could regulate the TA.

## 4. Material and methods

### 4.1. *Drosophila* stocks and culture condition

Fly stocks (electronic supplementary material, table S2) and crosses were maintained on standard *Drosophila* medium at 25°C. The flies were grown for 4 days at 29°C before dissection and fixation as described before [18]. For the temperature-shift studies, the stocks were maintained at 18°C and then shifted to 30°C for a limited period before dissection and fixation.

### 4.2. Whole-mount immunostaining

Testis from a 4-day-old male was dissected and fixed in 4% paraformaldehyde for 1 h at room temperature. The tissue was washed three times in 0.3% PTX (0.3% TritonX in 1× PBS), incubated with an appropriate dilution of primary antibody (Traffic jam 1 : 10 000, phospho-histone3 (Ser-10) 1 : 4000, dpERK 1 : 400, Armadillo 1 : 100) overnight, followed by washing and 2-h incubation at room temperature with Alexa dye-conjugated secondary antibody (1 : 400, Invitrogen). Counterstaining for visualization of nuclei was done with 0.001% Hoechst 33342 (Sigma Chemical Co., USA), then washed with 0.3% PTX and mounted in Vectashield*^®^* (Vector Laboratory Inc., USA).

### 4.3. Dye permeation assay

Adult testes were dissected in Schneider's medium, incubated in FITC-dextran solution and processed as described before [25].

### 4.4. Image acquisition, analysis and cyst profile quantification

All images were acquired using either an Olympus FV1000SPD laser scanning confocal microscope using 10×, 0.3 NA and 60×, 1.35 NA objectives. Some images were also acquired using the Zeiss 510meta laser scanning confocal microscope using the 63×, 1.4 NA objective. Multiple optical slices were collected covering the entire apical part of the testes. The images were analysed using ImageJ^®^ (http://fiji.sc/Fiji). The Cell-counter^TM^ plugin was used for counting the stained nuclei. The germ–soma ratio was estimated according to an earlier study by our group [31]. Briefly, all the Hoechst 33342-stained, as well as the *tj>His-RFP*-marked nuclei, present in the apical approximately 100 µm region of the testes were counted. Germline pool was estimated by subtracting the number of His-RFP marked nuclei from that of the total. The *traffic jam* promoter also marked the hub cells, which are recognized by the characteristic clustering of nuclei at the testis apex (electronic supplementary material, figure S1). The SCC and the total pool were estimated without the hub nuclei. For comparative analyses, we considered the fold change between germ–soma ratios of the genetically modified backgrounds and that of the His-RFP (control). It provided a quick quantitative estimate of the phenotype. Subsequently, we deployed a secondary screen using PH3 and Arm staining of the *vasa-GFP tjGal4>UAS-His2A-RFP/+*, *bamp-BamGFP tjGal4>UAS-His2A-RFP/+* and other suitably marked testes carrying different UAS-transgenes in the backgrounds. All scale bars, if not mentioned otherwise on the panels, measure 20 µm, the error bars depict ±s.d. and the pairwise significance of differences (*p*-values, *<0.05, **<0.01 and ***<0.001) for all histograms were estimated using Mann–Whitney *U*-test, whereas for all dot plots one-way ANOVA was applied.

## Supplementary Material

Suppelmentary Information

## Data accessibility

This article has no additional data.
