# Supplementary material for: Somatic ERK activation during transit amplification is essential for maintaining the synchrony of germline divisions in Drosophila testis
Source: Open Biol. 2018 Jul 25;8(7):180033. doi: 10.1098/rsob.180033 (PMC6070716; doi:10.1098/rsob.180033)
Supplement: Suppelmentary Information [file rsob180033supp1.pdf]

Supplementary information

Authors: Samir Gupta, Bhavana Varshney, Shambhabi Chatterjee and Krishanu Ray*

Title: Somatic ERK activation during the transit amplification is essential for maintaining the synchrony of germline divisions in *Drosophila* testis

Journal: Open Biology

Article DOI: 10.1098/rsob.20160049

Content:

Item	*Description*	*Page*
Figure S1	Organization of somatic cell nuclei at the testis apex. (related to Figure 1)	2
Figure S2	Rac expression in SCCs is redundant for regulating the early germline population. (related to Figure 1)	3
Figure S3	Effects of somatic alterations of Rac-Rho cascade and EGFR/ERK and their effects on Bam expression, as well as the divisions and differentiation of germline. (related to Figure 3)	4
Figure S4	Somatic modulation of EGFR and downstream components alter germline proliferation. (related to Figure 4)	5
Figure S5	Persistent somatic downregulation of EGFR activity alter spermatogonial proliferation and β-catenin localization on somatic cyst boundary. (related to Figure 4)	6
Figure S6	Somatic modulation of Rac1 activity does not alter the quality of somatic encapsulation of cysts. (related to Figure 5)	7
Figure S7	Constitutive activation of Armadillo/β-Catenin in somatic cells does not alter the cyst distribution. (related to Figure 6)	8
Table S1	The nuclei counts and estimates of the germ:soma ratios due to somatic perturbations of the EGFR and downstream signalling components. (related to Figure 1)	9
Table S2	The nuclei counts and estimates of the germ:soma ratios due to somatic perturbations of the Wnt signalling components. (related to Figure 6)	9
Table S3	List of fly stocks used.	10

Supplementary information

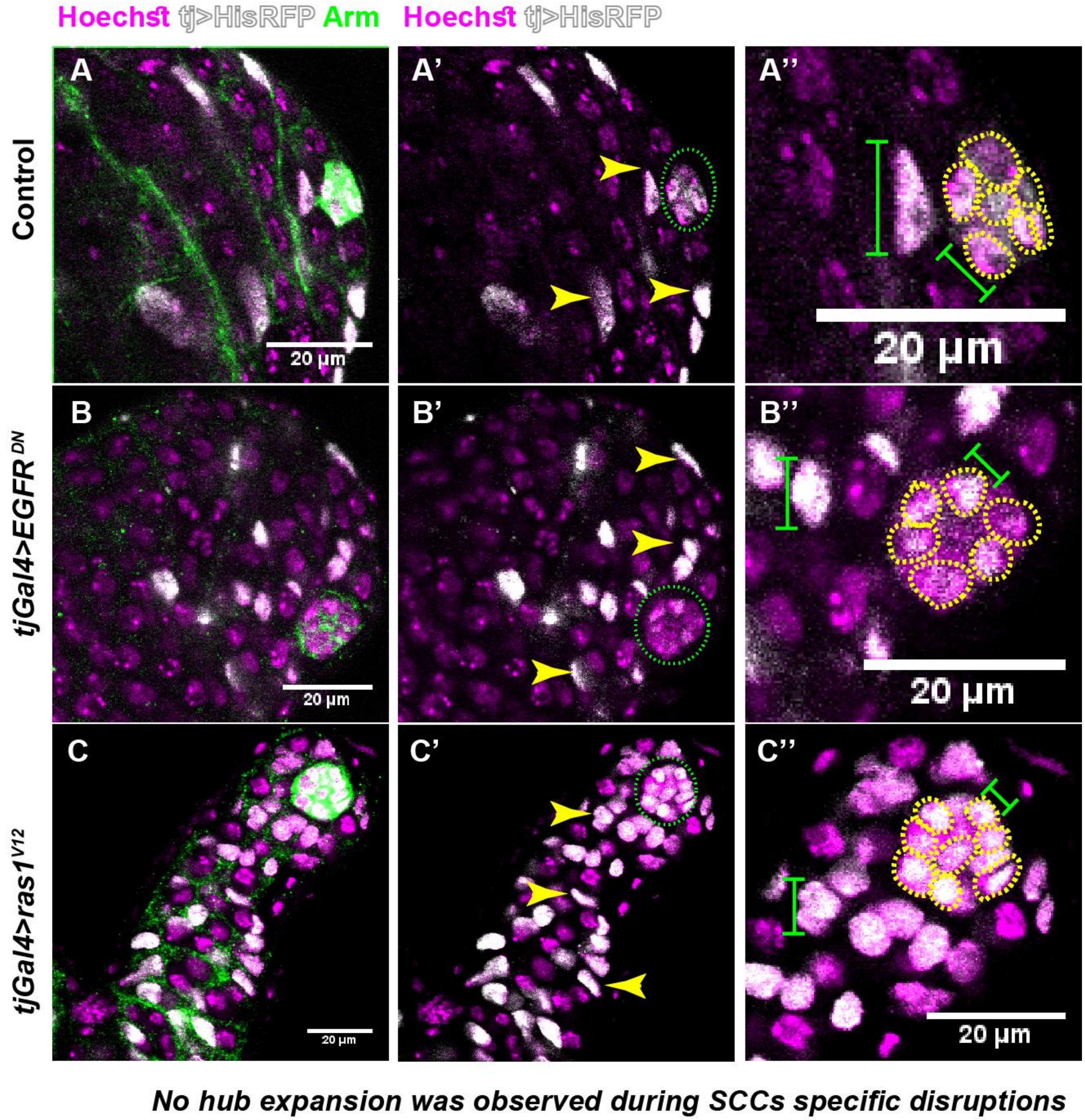

Figure S1 (related to Figure 1): Organization of somatic cell nuclei at the testis apex. A-C) Apical part of testes from *tjGal4/+; UAS-His2A-RFP/+* (Control) **(A-A'')**, *tjGal4/UAS-EGFR^{DN}; UAS-His2A-RFP/UAS-EGFR^{DN}* **(B-B'')**, and *tjGal4/+; UAS-His2A-RFP/UAS-ras85D^{V12}* **(C-C'')** immunostained with anti-Armadillo (green). His2A-RFP (white) expression marked the somatic cell nuclei, and all nuclei were stained with the Hoechst dye (magenta). Yellow arrow-heads point to early SCC nuclei **(A'-C')**, and green dotted circles depict the 'Hub' at the apical tips **(A'-C')**. Enlarged views of the testes apex **(A''-C'')** indicates characteristic nuclei organization in the Hub in control and mutant genotypes, respectively.

Supplementary information

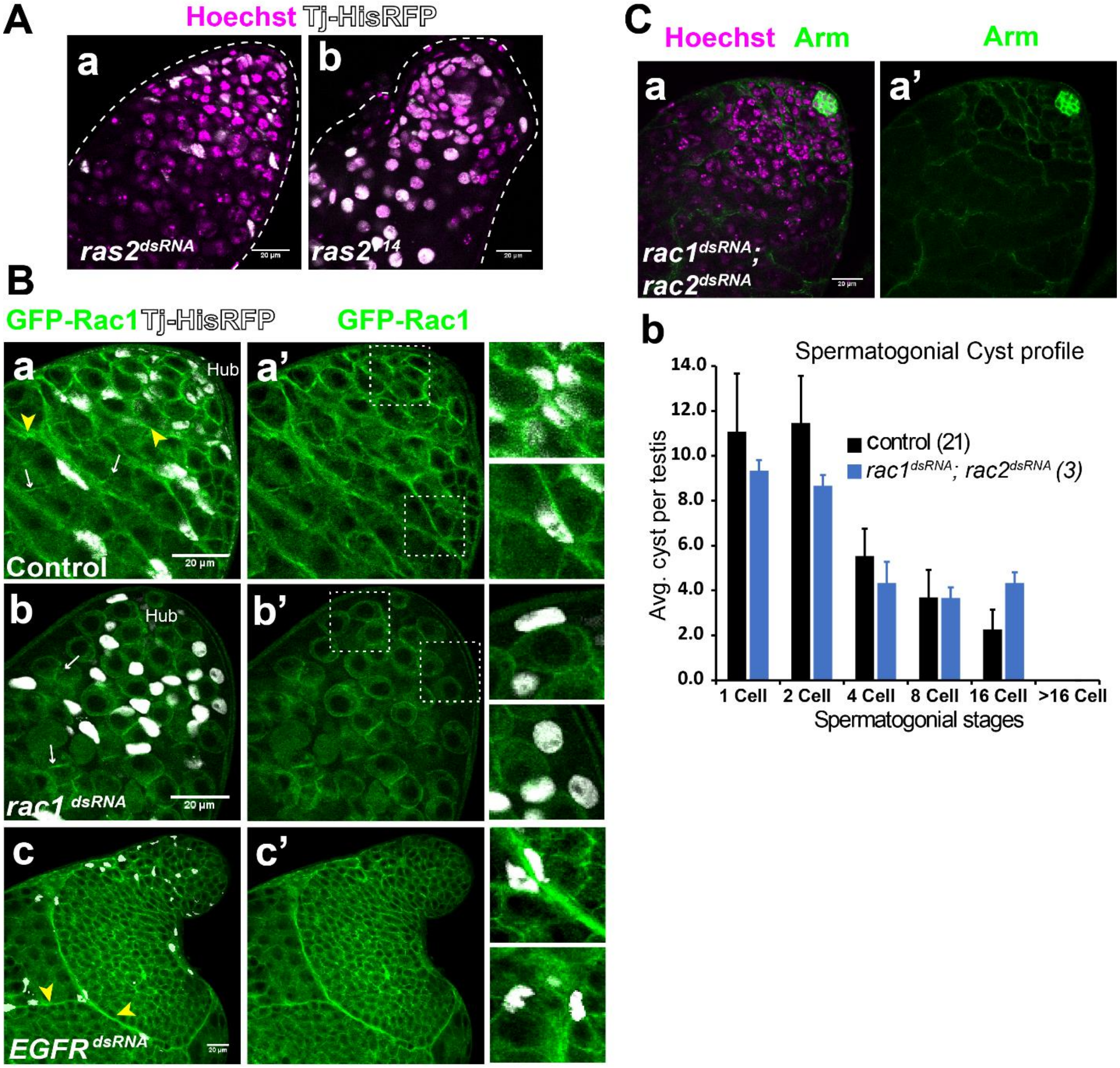

Figure S2 (related to Figure 1): Rac expression in SCCs is redundant for regulating the early germline population. A) Apical part of testes from *tjGal4/UAS-ras64D*^{dsRNA}*; UAS-HisRFP/+* (*ras2*^{dsRNA}) **(a)** and *tjGal4/+; UAS-HisRFP/UAS-ras64D*^{V14} (*Ras2*^{V14}) **(b)** males stained with Hoechst (magenta). His2A-RFP (white) expression marked the SCCs. **B)** Apical part of testes from *tjGal4/GFP-Rac1; UAS-His2A-RFP* (Control) **(a)**, *tjGal4/GFP-Rac1; UAS-rac1*^{dsRNA}*/UAS-His2A-RFP* (*rac1*^{dsRNA}) **(b)**, and *tjGal4/GFP-Rac1; UAS-EGFR*^{dsRNA}*/UAS-His2A-RFP* (*EGFR*^{dsRNA}) **(c)** depicts patterns of *Rac1-GFP* (green) and the *tj>HisRFP* (white) expressions and localizations. GFP-Rac1 localizations in the SCCs (yellow arrowheads) and at the interface of the germline cells (fine white arrows) are indicated. Enlarged views of the boxed regions in **a'-c'** are shown in the right panels. Note that the expression of *tjGal4>rac1*^{dsRNA} selectively eliminated GFP-Rac1 from the somatic cells without affecting the localizations at the germline interface **(b-b')**. Also, expression of *tjGal4>EGFR*^{dsRNA} did not disrupt the GFP-Rac1 localizations in SCCs **(**yellow arrowheads, **c-c')**. **C)** Apical part of testes from *tjGal4/UAS-rac2*^{dsRNA}*; UAS-rac1*^{dsRNA}*/+* (*rac1*^{dsRNA}*; rac2*^{dsRNA}) males stained with anti-Arm (green) and the Hoechst dye (magenta) **(a-a')**, Histograms **(b)** depict Cyst profile in control and *tjGal4/ UAS-rac2*^{dsRNA}*; UAS-rac1*^{dsRNA}*/+* backgrounds. Scale bars measure 20 µm**,** error bars depict $\pm$ S.D., and the pairwise significance of differences (p-values, * <0.05, **<0.01, and, ***<0.001) were estimated using one-way ANOVA® and Mann-Whitney U-test.

Supplementary information

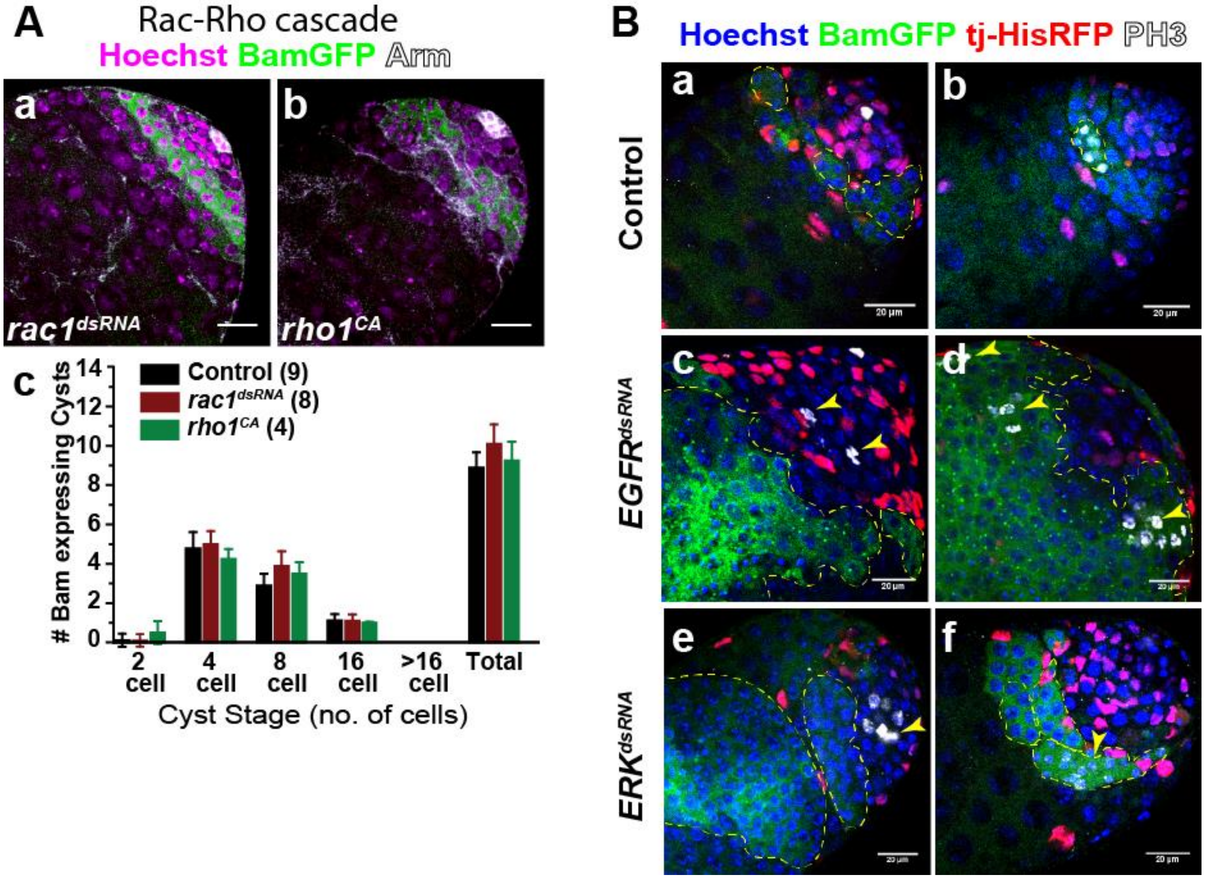

Figure S3 (related to Figure 3): Effects of somatic alterations of Rac-Rho cascade and EGFR/ERK and their effects on Bam expression, as well as the divisions and differentiation of germline. A) Testes from *tjGal4 /BamPbamGFP; UAS-rac1*^{dsRNA}*/UAS-His2A-RFP* **(a)** and, *tjGal4 /BamPbamGFP; UAS-rho*^{V14}*/UAS-His2A-RFP* **(b)** were immunostained with Arm (white) marking the cyst boundary and Hoechst dye (magenta), BamGFP (green) depicts bam reporter expression. **c)** Histograms depict the distribution of *bam*-expressing cysts in different genotypic backgrounds. **B)** Testes from *tjGal4/BamPbamGFP; UAS-His2A-RFP/+* **(a, b)**, *tjGal4/BamPbamGFP; UAS-His2A-RFP/EGFR*^{dsRNA} **(c, d)** and *tjGal4/BamPbamGFP; UAS-His2A-RFP/ERK*^{dsRNA} **(e, f)** were stained with anti-PH3 (grey), and Hoechst (magenta), HisRFP (red) marks SCCs nuclei, and BamGFP (green) depicts bam reporter expression. A broken yellow line marks individual cyst boundaries, and yellow arrowheads indicate individual and clusters of PH3-positive nuclei. Note that somatic knockdown of EGFR and ERK induces scattered mitoses within a cyst. Scale bars measure 20 µm**,** error bars depict $\pm$ S.D.

Supplementary information

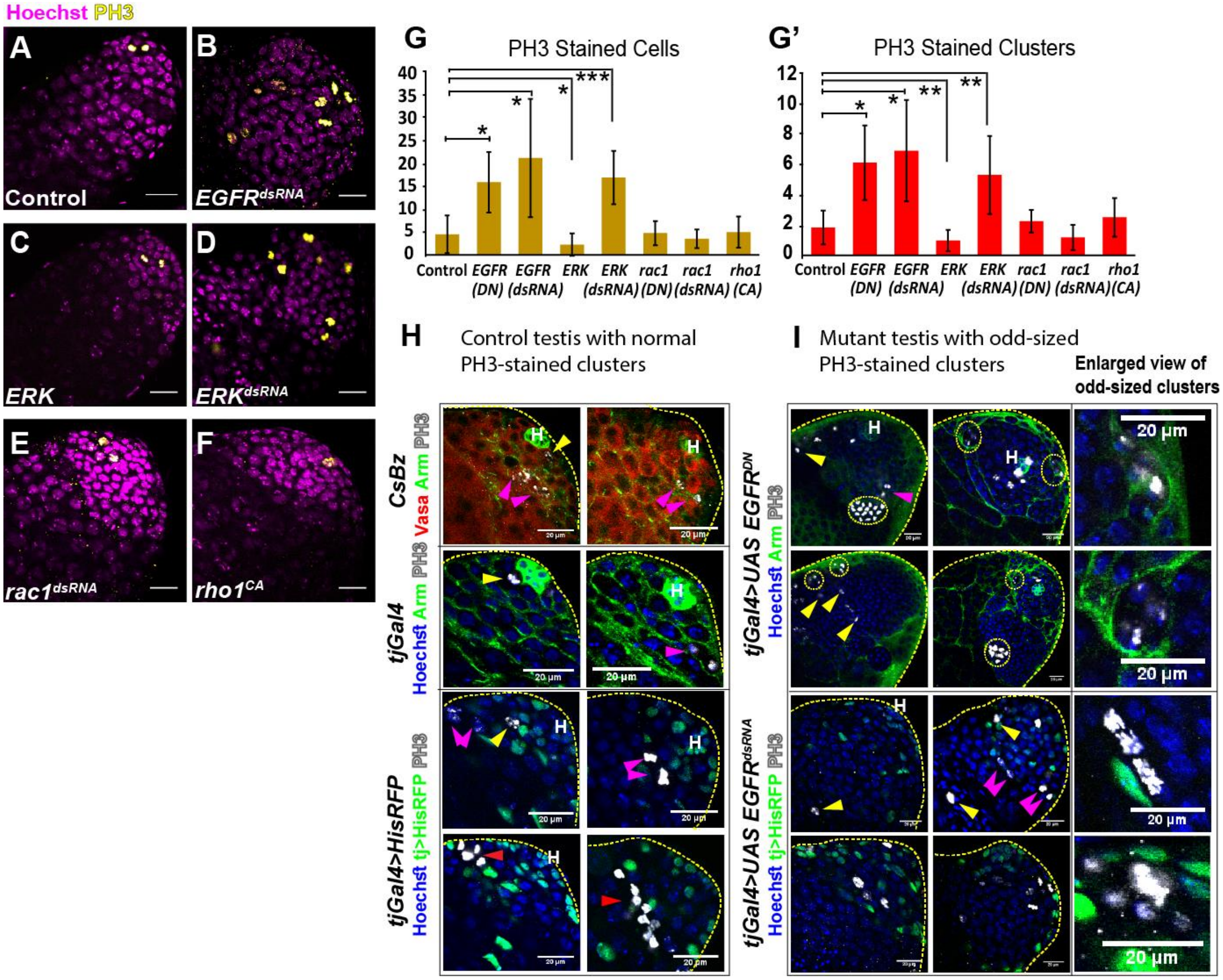

Figure S4 (related to Figure 4): Somatic modulation of EGFR and downstream components alter germline proliferation. A-F) Testes from *tjGal4/+; UAS-His2A-RFP/+* (A), *tjGal4/+; UAS-His2A-RFP/UAS-EGFR^{dsRNA}* (B), *tjGal4/+; UAS-His2A-RFP/UAS-ERK^{wt}* (C), *tjGal4/+; UAS-His2A-RFP/UAS-ERK^{dsRNA}* (D), *tjGal4/+; UAS-His2A-RFP/UAS-rac1^{dsRNA}* (E) and, *tjGal4/+; UAS-His2A-RFP/ UAS-rho1^{V14}* were immunostained with anti-PH3 (yellow) and the Hoechst dye (magenta). **G-G')** Histograms depict mean (±S.D.) PH3-stained cells (M-Phase index) and clusters respectively, in different genotypic backgrounds. **H-I)** Testes from control and mutant genotypes immunostained with PH3 (white), vasa (red), or arm (green) were stained for Hoechst (blue), HisRFP (green) marks SCCs nuclei, wherever shown. Enlarged views of PH3-stained clusters identifies odd-sized clusters in mutant genotypes. Scale bars measure 20 µm, error bars depict ± S.D., and the pairwise significance of differences (p-values, * <0.05, **<0.01, and, ***<0.001) were estimated using one-way ANOVA® and Mann-Whitney U-test.

Supplementary information

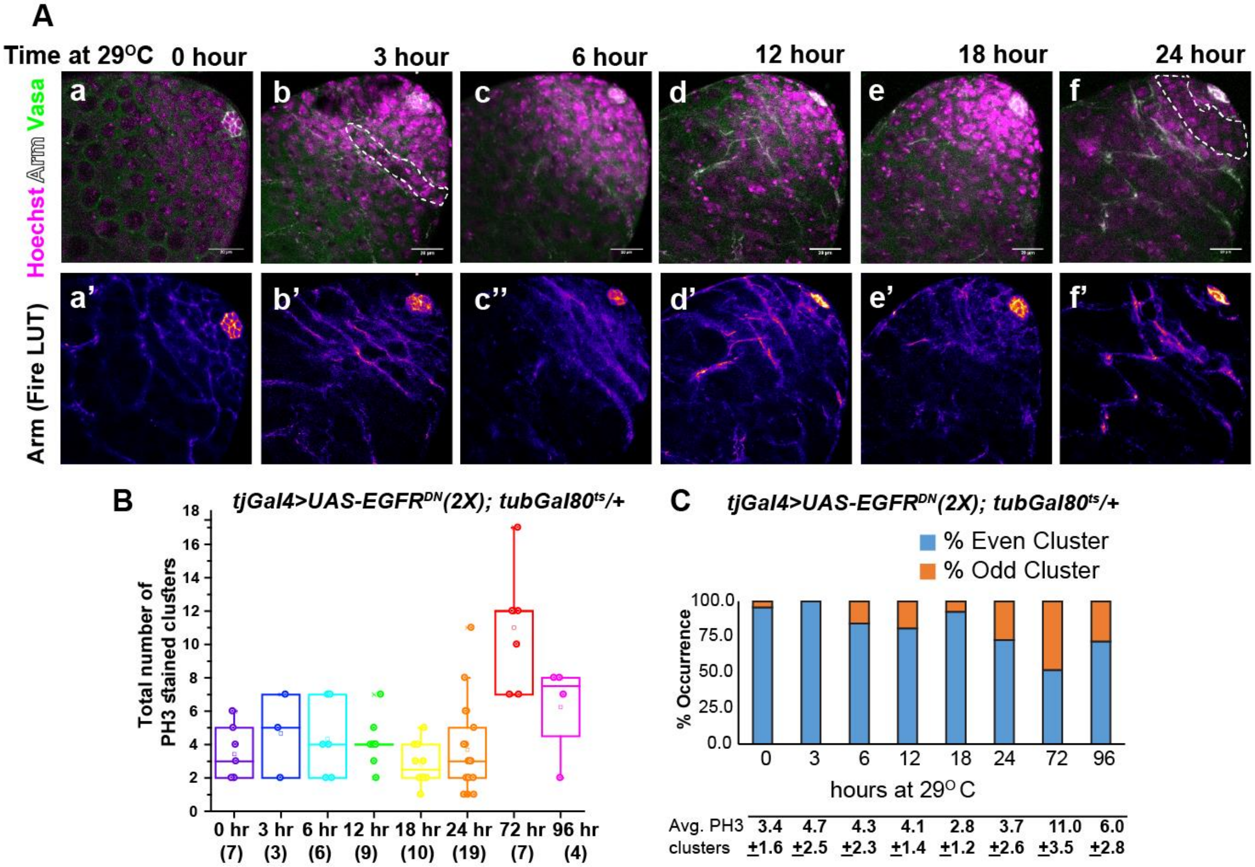

Figure S5 (related to Figure 4): Persistent somatic downregulation of EGFR activity alter spermatogonial proliferation and β-catenin localization on somatic cyst boundary. A) Testes from freshly eclosed flies of genotype *tjGal4/ UAS-EGFR^{DN}; tubGal80^{ts}/ UAS-EGFR^{DN}* that were allowed to age at 29℃ for different periods **(a-f)** as indicated on the top, were immunostained with Armadillo (white and FIRE LUT), Vasa (green), and the Hoechst dye (magenta). The scale bars indicate 20μm, broken white line marks a 16-cell cyst, and Arm staining is separately shown in the false color heat map (FIRE, ImageJ®) **(a'-f')**. Note that the uniformity of Arm staining is gradually disrupted with increasing duration of growth at the non-permissive temperature (29°C) which would proportionally increase the levels of EGFR^{DN} in somatic cyst cells. **B)** Box-plots depicts the occurrence of PH3-stained clusters in *tjGal4>EGFR^{DN} (2x) tubgal80^{ts}* testes after a certain duration of growth at 29°C (x-axis), which causes progressive inactivation of the somatic EGFR activity. The sample sizes are indicated in parenthesis below each box. **C)** Histograms depict relative occurrence of odd and even-sized PH3-stained clusters with decreasing dose of EGFR activity in SCC**.** Scale bars measure 20 μm, error bars depict ± S.D. Even though the total number of mitotic clusters does not increase significantly until 24 hours of growth, the probability of finding the odd-sized clusters appreciates to ~25% at this stage. The large (>16 cell) cysts appear in the testis after 24 hours of growth at the non-permissive temperature but the frequency of such occurrence is very low.

Supplementary information

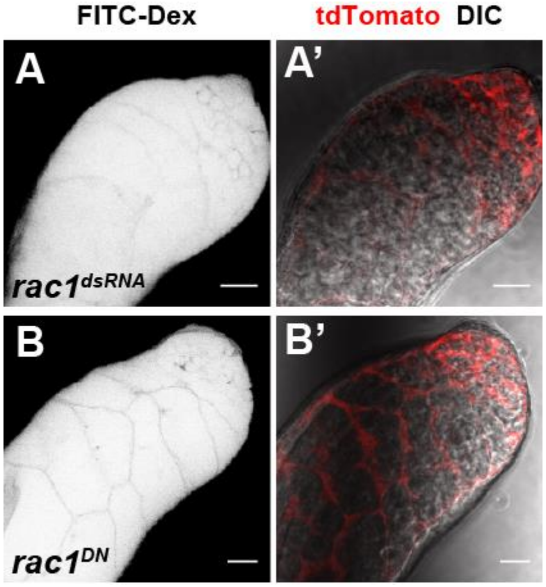

Figure S6 (related to Figure 5): Somatic modulation of Rac1 activity does not alter the quality of somatic encapsulation of cysts. A-B) Testes from *tjGal4/+; UAS-tdTomato/UAS-rac1*^{dsRNA} **(A)** *and tjGal4/+; UAS-tdTomato/UAS-rac1*^{DN} **(B)** were dissected in Schneider's medium and incubated in FITC-dextran (inverted LUT). Scale bars indicate 20 µm.

Supplementary information

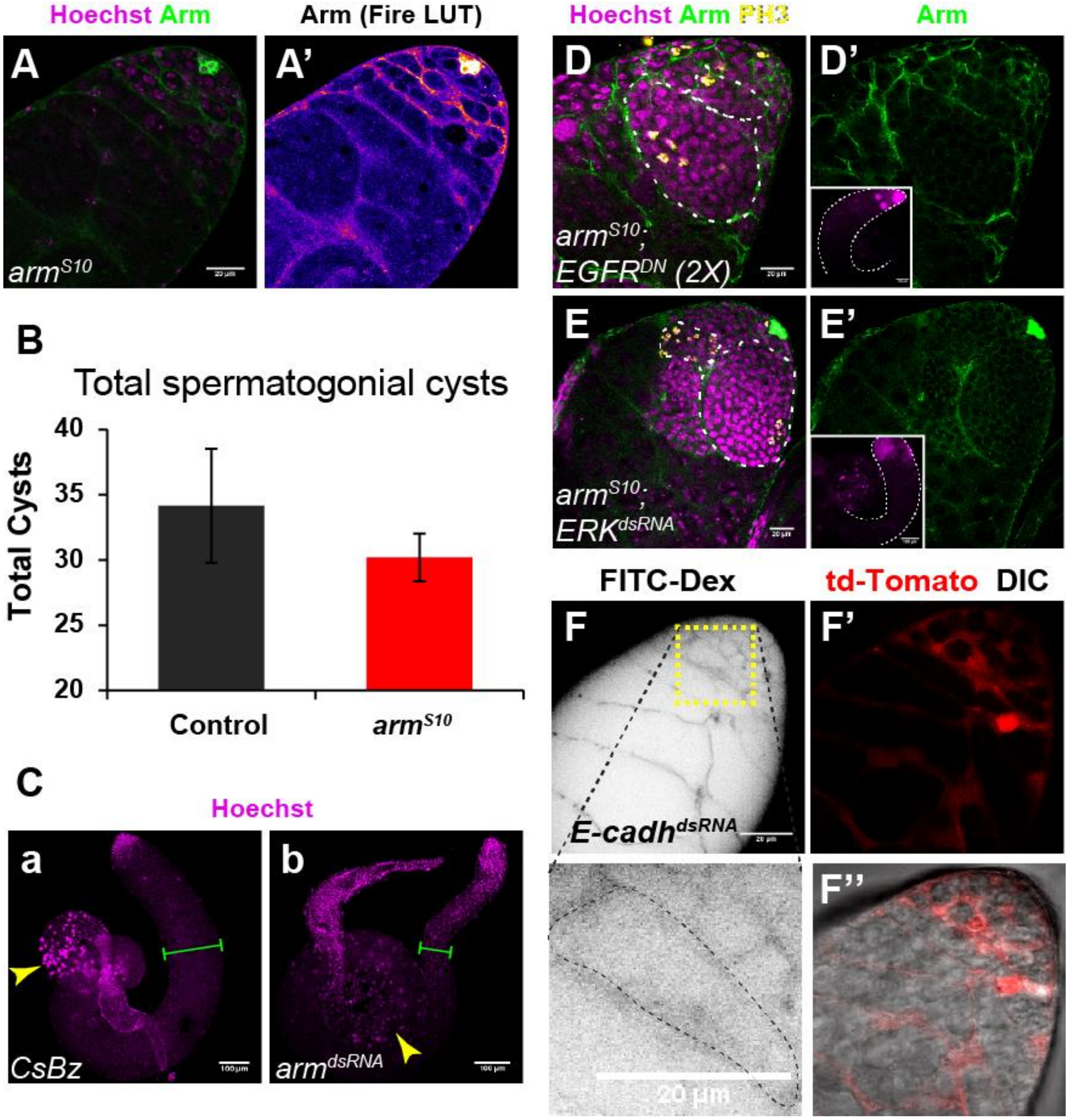

Figure S7 (related to Figure 6): Constitutive activation of Armadillo/β-Catenin in somatic cells does not alter the cyst distribution. A) Testis from *UAS-Arm^{S10}/Y; tjGal4/+; UAS-His2A-RFP/+* were immunostained with Arm (green) and the Hoechst dye (magenta). **B)** Histogram depicts cyst profile in control and *UAS-arm^{S10}/Y; tjGal4/+* genetic backgrounds. **C)** Testis from *CsBz* (Wild-type) **(C-a')** and *tjGal4/+; UAS-arm^{dsRNA}*/UAS-His2A-RFP **(C-b')** were stained with the Hoechst dye (magenta). *arm^{dsRNA}* testis were visibly thinner. Arrowhead (yellow) point to the end of the testis. Note that the mature nuclei bundles at the base are much less in the Arm RNAi background. **D-E)** Testis from *UAS-Arm^{S10}/Y; tjGal4/UAS-EGFR^{DN}; UAS-His2A-RFP/UAS-EGFR^{DN}* **(D)** and Testis from *UAS-Arm^{S10}/Y; tjGal4/+; UAS-His2A-RFP/UAS-ERK^{dsRNA}* **(E)** were immunostained with Arm (green) and PH3 (yellow), and the Hoechst dye (magenta). **D'-E')** anti-Arm staining is depicted in green. **F)** Testes from *tjGal4/+; UAS-tdTomato/UAS-E-cadh^{dsRNA}* were dissected in Schneider's medium and incubated in FITC-dextran (inverted LUT). **(C)**, and Insets in **(D')** and **(E')** have scale bar of 100 µm whereas other scale bars, error bars, and statistical significance are applied according to the description in Fig S3.

Supplementary information

Table S1 (related to Figure 1): The nuclei counts and estimates of the germ:soma ratios due to somatic perturbations of the EGFR and downstream signalling components.

N	Transgene	# Nuclei (Avg ± SD)			Avg. Germ/ Soma ratio ± SD	Fold Change in Ratio ± SD
		Total	Germline	Somatic		
21	*HisRFP*	324.6 ± 27.8	261 ± 24.8	63.6 ± 5.1	4.1 ± 0.3	1.0 ± 0.1
12	*$EGFR^{DN}$ (2X)*	789.8 ± 162.7	726.9 ± 152.3	62.8 ± 19.4	12.5 ± 3.8	3.0 ± 0.9
6	*$EGFR^{dsRNA}$*	712 ± 39.5	662.6 ± 48.2	49.4 ± 17.9	15.3 ± 6.8	3.7 ± 1.6
6	*$EGFR^{WT}$*	299.2 ± 54.4	200.5 ± 42.1	98.7 ± 15.4	2.0 ± 0.3	0.5 ± 0.1
7	*sSpitz*	248.7 ± 29.9	167.6 ± 17.4	81.1 ± 16.2	2.1 ± 0.3	0.5 ± 0.1
9	*$spitz^{dsRNA}$*	286.6 ± 36	234.6 ± 29.3	52.0 ± 7.1	4.5 ± 0.2	1.1 ± 0.0
10	*$ras1^{DN}$*	306.7 ± 16.5	259.6 ± 13.9	47.1 ± 3.8	5.5 ± 0.3	1.3 ± 0.1
7	*$Ras1^{dsRNA}$*	314.4 ± 30.2	259.9 ± 13.9	54.6 ± 10	4.9 ± 0.8	1.2 ± 0.2
9	*ERK^{WT}*	329.9 ± 27.8	262.6 ± 22.8	67.3 ± 5.7	3.9 ± 0.2	0.9 ± 0.0
8	*ERK^{dsRNA}*	516.0 ± 21.4	458.6 ± 19.9	57.4 ± 10.7	8.3 ± 2.0	2.0 ± 0.5
10	*$ras1^{V12}$*	146.7 ± 32.1	88.5 ± 31.8	58.2 ± 18.1	1.7 ± 0.8	0.4 ± 0.2
6	*$ras1^{V12S35}$*	161.2 ± 27.7	79.2 ± 23.6	82.0 ± 27.3	1.1 ± 0.6	0.3 ± 0.1
7	*$basket^{dsRNA}$*	417.7 ± 43.6	350.3 ± 46.0	67.4 ± 16.7	5.6 ± 1.8	1.4 ± 0.4
10	*vav^{dsRNA}*	276.7 ± 77.7	231.4 ± 68.2	45.3 ± 14.8	5.3 ± 1.6	1.3 ± 0.4
14	*$rac1^{DN}$*	461.6 ± 103.4	406.1 ± 104.7	55.5 ± 11.1	7.6 ± 2.6	1.9 ± 0.6
12	*$rac1^{dsRNA}$*	429.1 ± 82.1	372.3 ± 78.9	57.3 ± 18.3	7.1 ± 2.6	1.7 ± 2.6
11	*$rac2^{dsRNA}$*	598.8 ± 37.9	515.5 ± 41.0	83.3 ± 6.2	6.2 ± 1.0	1.9 ± 0.6
10	*$rho1^{WT}$*	549.1 ± 37.9	470.8 ± 96.6	78.3 ± 12.3	6.0 ± 0.7	1.5 ± 0.2
21	*$rho1^{CA}$*	453.1 ± 89.7	372.9 ± 84.1	80.2 ± 36.2	5.6 ± 2.6	1.4 ± 0.6

Table S2 (related to Figure 6): The nuclei counts and estimates of the germ:soma ratios due to somatic perturbations of the Wnt signalling components.

N	Transgene	# Nuclei (Avg ± SD)			Avg. Germ/ Soma ratio ± SD	Fold Change in Ratio ± SD
		Total	Germline	Somatic		
10	*arm^{dsRNA}*	418.5 ± 25.4	348.4 ± 24.5	70.1 ± 1.7	5.0 ± 0.3	1.2 ± 0.1
6	*arm^{S10}*	318.2 ± 13.2	240 ± 11	78.2 ± 3.1	3.1 ± 0.1	0.7 ± 0.0
4	*$DTCF^{DN}$*	334.3 ± 11	275.3 ± 10.6	59 ± 1.6	4.7 ± 0.2	1.1 ± 0
4	*$shaggy^{DN}$*	317.5 ± 6.6	232.5 ± 7.3	85 ± 6.1	2.7 ± 0.2	0.7 ± 0.1
8	*$shaggy^{CA}$*	386.1 ± 14.2	313.3 ± 13.2	72.9 ± 2	4.3 ± 0.2	1.04 ± 0.04

Supplementary information

Table S3: List of fly stocks used.

Fly stock	Description	Reference (Source)
tjGal4/Cyo	Gal4 expression under *traffic jam* promoter	Tanentzapf, G., Devenport, D., Godt, D. & Brown, N. H. *Nat Cell Biol* **9**, 1413-1418 (2007). (DGRC, Kyoto; Dorothea Godt lab, Univ. of Toronto)
nosGal4 vp16/Tm3	Gal4 expression under nanos promoter	P. Rorth, Mech. Dev. 78, 113-118 (1998).
bamGal4/Cyo	Gal4 expression under bag-of-marbles promoter	Chen, D. & McKearin, D. M. *Curr. Biol.* **13**, 1786-1791 (2003). (McKearin and Buszczak labs, UT Southwestern
UAS-HisRFP	Expresses Histone 2Av tagged to RFP under UAS control	Heidmann, 2007.2, P{His2Av-mRFP1} insertions.<br>Bloomington Stock 23650
bamP-bamGFP	Bam-GFP fusion protein expressed under 5'-*bam* promoter, mimic endogenous *bam* expression pattern	Chen, D. & McKearin, D. M. *Curr. Biol.* **13**, 1786-1791 (2003). (McKearin and Buszczak labs, UT Southwestern)
GFP-Rac1	RacGFP fusion protein under rac1 promoter	Abreu-Blanco *et al*., Curr. Biol. 24, 144-155 (2014). doi.org/10.1016/j.cub.2013.11.048<br>Bloomington stock 52284
UAS-Egfr^{CA}	Overexpression of constitutively activated human EGFR, ligand-independent under UAS control	Yang, L & Baker, N. BMC Dev. Biol. **6**,1-8 (2006)<br>Bloomington stock 9534
UAS-EGFR^{dsRNA}	Double-stranded RNA against EGFR mRNA for interference effecting in knockdown	Ni *et al*., 2011.7.19, TRiP germline vectors pVALIUM20, pVALIUM21 and pVALIUM22<br>Bloomington 36770, TRiP Line
UAS-Egfr^{WT}	Overexpression of mammalian EGFR1 under UAS control	Yang, L & Baker, N. BMC Dev. Biol. **6**,1-8 (2006)<br>Bloomington stock 9533
UAS-Egfr^{DN}	Expresses Dominant negative form of EGFR (dimerization competent but signalling incompetent) under UAS control	Buff, E., Carmena, A., Gisselbrecht, S., Jimenez, F. & Michelson, A. M. *Development* **125**, 2075-2086 (1998)<br>(*Drosophila* stock center, Bloomington, Indiana, USA)
UAS-spi^{dsRNA}	Double-stranded RNA against spitz mRNA for interference effecting in knockdown	Ni *et al*., 2010.12.1, A genome-scale shRNA resource for transgenic RNAi in *Drosophila*.
UAS-sspi	Expression of the secreted form of spitz under UAS control	Rorth, 2014.11.24, spi insertion from Pernille Rorth.<br>spi insertion from Pernille Rorth
spi$^{77-20}$	Temperature sensitive allele of Spitz	Isolated by Wakimoto and Zuker (1995), reported in Sarkar *et al*., Curr Biol, 17, 1253-1254 (2007).
UAS-argos	Expression of the secreted form of EGF antagonist argos under UAS control	Bloomington Stock BL-5363<br>Michelson, 1999.8.9, UAS constructs and insertions from Alan Michelson.<br>UAS constructs and insertions from Alan Michelson
UAS-ras85D^{DN}	Expresses Dominant negative form of Ras under UAS control	Montell, 1998.11.25, Insertions of ras and ral constructs.<br>Insertions of ras and ral constructs

Supplementary information

Table S3: continued

Fly stock	Description	Reference (Source)
UAS-ras85D^{dsRNA}	Double-stranded RNA against ras85D mRNA for interference effecting in knockdown	Ni *et al*., 2010.12.1, A genome-scale shRNA resource for transgenic RNAi in *Drosophila*. Bloomington stock
UAS-ras85D^{V12}<br>*UAS-ras85D*^{V12S35}<br>*UAS-ras85D*^{V12C40}<br>*UAS-ras85D*^{V12G37}	Ras effector loop mutations for constitutive activation of ras and further specific downstream activation	Karim & Rubin 125(1), 1-9 (1998). Gift from K. Irvine Lab
UAS-rolled^{WT}	Overexpression of wild-type Rolled (ERK)	Moses, 2011.7.29, P{UAS-rl.K}2A. Bloomington stock 36270
UAS-rolled^{dsRNA}	Double-stranded RNA against mRNA for interference effecting in knockdown	Ni *et al*., 2011.7.19, TRiP germline vectors pVALIUM20, pVALIUM21, and pVALIUM22. Bloomington stock 34855
UAS-Basket^{dsRNA}	For tissue-specific expression of dsRNAs using the Gal4/UAS system	Rallis *et al*., 2010, Dev. Biol. 339(1): 65-77 VDRC ID: 34139
UAS-rho^{dsRNA}	Expresses dsRNA for RNAi of Rho1 under UAS control	Perkins *et al*., 2009, Update to the TRiP stock collection Bloomington stock 27727
UAS-rac1^{dsRNA}	Expresses dsRNA for RNAi of Rac1 under UAS control	Perkins *et al*., 2009, Update to the TRiP stock collection. Bloomington stock 28985
UAS-vav^{dsRNA}<br>*UAS-rac2*^{dsRNA}<br>*UAS-vn*^{dsRNA}<br>*UAS-arm*^{dsRNA}	For tissue-specific expression of dsRNAs using Gal4/UAS system; all inserts have been molecularly validated by VDRC	Dietzl *et al.*, 2007, *Nature* 448, 151-156. (VDRC, Austria) (http://stockcenter.vdrc.at/control/main)
UAS-rho1^{WT}	Expresses wild-type Rho1 under UAS control	Warner, 2010.9.15, Warner construct and insertions. Bloomington stock 28872
UAS-rho^{dsRNA}	Expresses dsRNA for RNAi of Rho1 under UAS control	Perkins *et al*., 2009, Update to the TRiP stock collection Bloomington stock 27727
UAS-rac1^{dsRNA}	Expresses dsRNA for RNAi of Rac1 under UAS control	Perkins *et al*., 2009, Update to the TRiP stock collection. Bloomington stock 28985
UAS-dlg1^{dsRNA}	Expresses dsRNA for RNAi of Rac1 under UAS control	Perkins et al., 2009, Update to the TRiP stock collection. Bloomington stock 25780
UAS-nrx^{dsRNA}	For tissue-specific expression of dsRNAs using Gal4/UAS system; all inserts have been molecularly validated by VDRC	Dietzl *et al.*, 2007, *Nature* 448, 151-156. (VDRC, Austria) (http://stockcenter.vdrc.at/control/main) VDRC: v8353
UAS-ECadh^{dsRNA}	Expresses dsRNA for RNAi of Rac1 under UAS control	Ni et al, 2010. A genome-scale shRNA resource for transgenic RNAi in Drosophila. Bloomington stock 38207

Supplementary information

Table S3: continued

Fly stock	Description	Reference (Source)
UAS-vav^{dsRNA}<br>*UAS-rac2*^{dsRNA}<br>*UAS-vn*^{dsRNA}<br>*UAS-arm*^{dsRNA}	For tissue-specific expression of dsRNAs using Gal4/UAS system; all inserts have been molecularly validated by VDRC	Dietzl *et al.*, 2007, *Nature* 448, 151-156.<br>(VDRC, Austria)<br>(http://stockcenter.vdrc.at/control/main)
UAS-rho1^{WT}	Expresses wild-type Rho1 under UAS control	Warner, 2010.9.15, Warner construct and insertions.<br>Bloomington stock 28872
UAS-rho1^{CA}	Expresses a constitutively-active Rho1 under the control of UAS	Weber and Mlodzik, 2003.11, UAS-Rho1 insertions.<br>Bloomington Stock 8144
UAS-arm^{S10}	Expresses stable form of β-catenin	Bloomington Stock 4782<br>Morel and Arias, 2004, Development 131(14): 3273--3283
UAS-DTCF^{DN}	Expresses suppressor of pangolin	Bloomington stock 4784<br>Donor: Mark Peifer, University of North Carolina, Chapel Hill
UAS-tdtomato/Tm6	Tandem dimer of tomato fluorophore under UAS promoter	Bloomington 32221<br>Donor: Gerald M. Rubin & Barret Pfeiffer, Howard Hughes Medical Institute, Janelia Research Campus
